# Novel millimeter-wave-based method for *in situ* cell isolation and other applications

**DOI:** 10.1038/s41598-018-32950-w

**Published:** 2018-10-03

**Authors:** Barney Boyce, Natalia Samsonova

**Affiliations:** 1 In Vivo Scientific, LLC 5 Gybe Ho Ct, Salem, SC 26976 USA; 2 CellEraser, LLC 15649 Century Lake Dr., Chesterfield, MO 63017 USA

## Abstract

As an alternative to laser-based methods, we developed a novel *in situ* cell isolation method and instrument based on local water absorption of millimeter wave (MMW) radiation that occurs in cellular material and nearby culture medium while the cultureware materials (plastic and glass) are transparent to MMW frequencies. Unwanted cells within cell population are targeted with MMWs in order to kill them by overheating. The instrument rapidly (within 2–3 seconds) heats a cell culture area of about 500 µm in diameter to 50 °C using a low-power W-band (94 GHz) MMW source. Heated cells in the area detach from the substrate and can be removed by a media change leaving a bare spot. Hence we named the instrument “CellEraser”. Quick, local and non-contact heating with sharp boundaries of the heated area allows elimination of the unwanted cells without affecting the neighboring cells. The instrument is implemented as a compact microscope attachment and the selective hyperthermic treatment can be done manually or in an automated mode. Mammalian cells heated even momentarily above 50 °C will not survive. This “temperature of no return” does not compromise cellular membranes nor does it denature proteins. Using the CellEraser instrument we found that the key event that determines the fate of a cell at elevated temperatures is whether or not the selectivity of its nucleus is compromised. If a cell nucleus becomes “leaky” allowing normally excluded (cytoplasmic) proteins in and normally nuclear-localized proteins out, that cell is destined to die. Quick heating by MMWs to higher temperatures (70 °C) denatures cellular proteins but the cells are not able to detach from the substrate – instead they undergo a phenomenon we called “thermofixation”: such cells look similar to cells fixed with common chemical fixatives. They remain flat and are not washable from the substrate. Interestingly, their membranes become permeable to DNA dyes and even to antibodies. Thermofixation allows the use of western blot antibodies for immunofluorescence imaging.

## Introduction

Existing methods for targeted elimination of anchorage-dependent cells generally utilize laser technology such as laser ablation and laser microdissection^1,2^. In these methods a high power laser beam (usually from a pulsed UV laser) is used to either sweep over the surface to lethally illuminate the unwanted cells^3^, or to cut out the cells of interest and physically separate them from the remaining cells^4,5^.

One promising cell isolation technology based on *in situ* photothermal laser processing was briefly commercialized in the 1980s^2,6^ but is no longer available. In this approach the cells were grown on a thin heat-absorptive adhering film. A laser was used either for direct cell irradiation and photothermal elimination or as a “cutter” when the desired cells were circumscribed with the laser to cut the film, followed by peeling of the film to remove undesired cells, leaving behind an island of desired cells on the surface. Thus the undesired cells were eliminated, and the selected cell population remained on the incised film disks adhered to the culture chamber where growth and proliferation can continue.

Another laser-based instrument for *in situ* cell isolation called LEAP™ (laser enabled analysis and processing) was commercialized by Cyntellect, Inc. in early 2000s^7^. Depending on the laser power and wavelength used, the instrument could irradiate and eliminate the unwanted cells either by photothermal or photochemical mechanisms. The phototermal approach required the presence of Allura Red AC dye in the cell culture to enhance the light energy absorption and resulted in immediate protein coagulation and cell necrosis. There are other examples of the use of laser irradiation in the presence of a chromophore to destroy cells *in vitro*^2^ and *in vivo*^8,9^. The dye-free photochemical approach used more laser power but did not lead to immediate destruction of targeted cells - instead it induced apoptosis over a period of 4 to 24 hous. The LEAP instrument is currently not on the market.

The only laser-based technique commercially available currently is a laser microdissection method, also known as LMD or LCM (laser capture microdissection)^4,5,10^. The LMD system is based on an upright microscope and focused laser is used for cutting plastic films where adherent cells are growing. The dissected piece of plastic with living cells on it falls by the force of gravity into a collection container where subsequent recultivation is possible as well as the downstream analysis of the extracted cells. The LMD is claimed by the manufacturers to be a contact- and contamination-free method for isolating specific single cells or areas of tissue from tissue samples.

The shortcoming of the laser ablation approach is that a lot of free radicals and products of oxidation are formed during the ablation process^11^. Also there is a leakage of cellular content into the culture medium. These aggressive byproducts harm the desired cells. The microdissection approach is not sterile and requires special consumables. All laser-based methods need special and expensive equipment, precise laser optics adjustment and in some cases, addition of special light energy absorbing dye to the cell media to intensify the cell damage. The majority of these methods cannot be used on a standard microscope and cannot be performed by an inexperienced operator.

As an alternative to LEAP or LMD techniques, we developed a novel *in situ* cell isolation method where selective hyperthermic damage is used to target and eliminate the unwanted cells^12^. The damage is done by local heating of adherent cell culture using millimeter wave (MMW) radiation. Cells heated above 50 °C round up, detach from the substrate and can be washed out by a media change as if the culture was “erased” – that is why the instrument is called CellEraser™. The CellEraser treatment could be repeated until all unwanted cells are removed from the culture without affecting the desired cells. Implemented as a compact microscope attachment, the instrument provides an easy, convenient, quick and inexpensive way for cell culture manipulation and cell isolation.

## Results

### CellEraser: method and device for selective hyperthermic damage of target cells

#### Principle and prototypes

The method^12^ employs millimeter waves - MMW, electromagnetic radiation with the half-wavelength of about a millimeter or about 150 GHz in frequency. We used MMWs in the W-band that ranges from 75 to 110 GHz. The method is based on unique properties of MMW radiation exhibited upon interaction with water. When a millimeter wave meets a water phase, it cannot penetrate into the bulk of the water. Instead, all the MMW energy is released as heat in a narrow surface layer – for example, 94 GHz MMW have water penetration depth of about 0.36 mm^13^. The frequency of 94 GHz was chosen as it is most efficiently absorbed by water (Supplementary Fig. 1), which makes this frequency most suitable for heating the surface layers of water-based cell culture mediums. Plastic and glass cultureware materials are transparent to MMW radiation (up to 300 GHz)^14,15^ so the cultureware bottom is not heated by MMWs.

**Figure 1:**
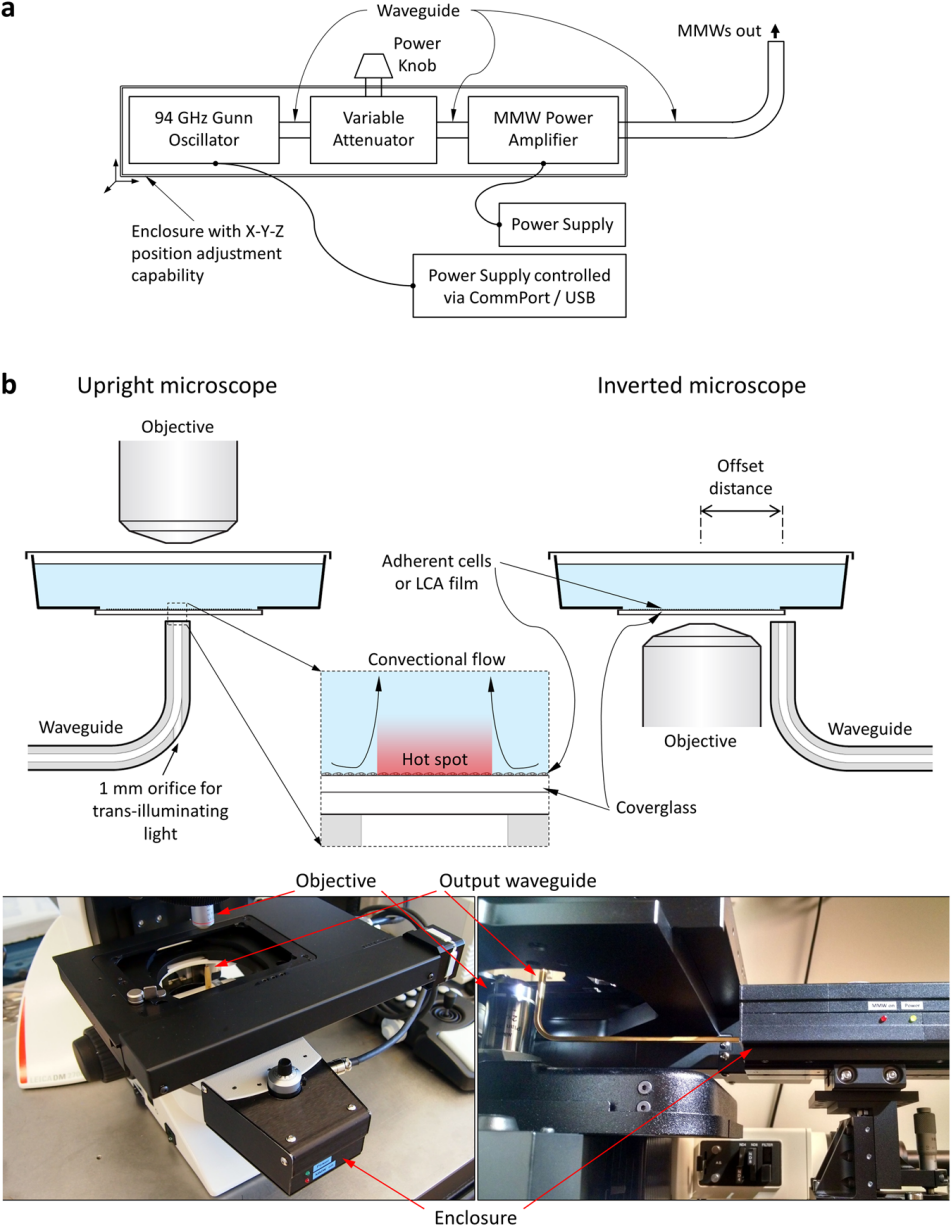
CellEraser instrument. (**a**) A diagram of the millimeter wave based device for selective hyperthermic damage of target cells (CellEraser). The device outputs a 3D positioned MMW radiation of a controlled power. (**b**) An illustration of CellEraser’s positioning and operation in the case of upright (left panel) and inverted (right panel) microscopes. Only the output waveguide is shown in both cases (see panel a). It is placed below the coverglass bottom of the cultureware to deliver the MMW radiation to a predetermined spot of a cell culture grown in the cultureware. The insert in the middle is a zoomed-in image of the end face of the waveguide and the coverglass bottom of the cultureware showing a spot of the culture medium heated by the MMW radiation. The upright microscope version of the device has an orifice of ~1 mm in diameter in the lower surface of the waveguide bend to provide for the trans-illumination, allowing direct observation of MMW application to the cell culture. In the inverted microscope version of CellEraser, imaging and MMW application have to be done sequentially as both the objective and the waveguide are located below the specimen: the target cells have to be moved between the objective position and the waveguide position separated by the “offset distance”. The pictures below show the upright microscope version of the instrument on a Leica DM2700 and the inverted version with Nikon Eclipse Ti.

Figure 1a shows a diagram of the device for selective hyperthermic damage of target cells. It includes a source of millimeter wave radiation – a fixed frequency Gunn oscillator (InP or GaN Gunn diode) that produces a stable output signal of 94 GHz with a maximum power output of 65–100 mW, a passive variable attenuator to control the radiation level, a narrow-band millimeter wave power amplifier to increase the millimeter wave radiation power to 250–400 mW or higher, and two power supplies to feed the Gunn oscillator and the amplifier. The on/off triggering is done by the power supply of the MMW source either manually or remotely via CommPort/USB for automatic operation. The MMW components are connected via waveguides containing the standing wave. The internal dimensions of the WR10 waveguide suitable for transmitting 94 GHz MMWs are 2.540 × 1.270 mm. The output waveguide has a 90 degree E-plane bend allowing its end face to be positioned in close proximity to the bottom of the cell cultureware. All elements of the device are placed in an enclosure. The 3D position of the enclosure can be adjusted to provide for position fine-tuning of the end face of the output waveguide.

The CellEraser can be used with any standard upright or inverted microscope (Fig. 1b). The end face of the output waveguide is positioned below the bottom of the cell cultureware to deliver the MMW radiation to a predetermined spot of a cell culture grown in the cultureware. Radiation power, exposure time of the MMW radiation, and positional relationship of the end face of the waveguide and of the target cells are selected such as to provide selective hyperthermic damage to the target cells. For best results, thin optical plastic or coverglass bottom cultureware should be used to shorten the distance between the cells and the end face of the output waveguide. Standard plastic cultureware (plates and flasks) also could be used but the “hot spot” in this case will have bigger size with less defined boundaries. The MMW radiation heats a narrow bottom layer of the culture medium where target adherent cells are located, without heating its bulk or the cultureware material (glass/plastic). Convection drives the heated media upwards towards the surface where heat dissipates (illustrated by applying MMWs to cell suspension, Supplementary Movie 1). The cells are kept sterile as the process does not require the cultureware lid removal.

The upright microscope version of the device (Fig. 1b, left panel) allows direct observation of MMW application to the cell culture. The output waveguide had to be modified with an orifice of ~1 mm in diameter in the lower surface of the waveguide bend to allow for the trans-illumination light to reach the cells through the vertical portion of the waveguide. The downside of this configuration is that a long distance objective has to be used to image the cells limiting the imaging capabilities significantly due to its low numerical aperture.

The advantage of the inverted microscope version of CellEraser (Fig. 1b, right panel) is that the output waveguide is not positioned in the optical path ruling out any interference with imaging. The vertical portion of the waveguide is moved away from the optical axis by an “offset distance” so that the waveguide goes in parallel to the objective and the center lines of the waveguide and the objective are separated by the offset distance. To expose the target cells to MMW radiation, the cells have to be moved to the waveguide position and then they have to be returned back to the objective position to see the aftermath of the exposure. An automated microscope stage is required to perform the process. The real-time observation of the MMW effect on the specimen is not possible in the inverted microscope configuration as the MMW application and imaging have to be done sequentially.

The lower part of Fig. 1b pictures the prototype instruments for upright (Leica DM2700) and inverted (Nikon Eclipse Ti) microscopes. For the upright microscope the device is mounted in place of the condenser utilizing 3D position adjustment capabilities of the condenser. In the case of an inverted microscope, CellEraser is supported by a position-adjustable stand.

#### Calibration

The calibration procedure is performed to ensure that the millimeter wave radiation level is high enough to heat the cells in the region of exposure to a desired temperature. To do that, a thin film (about 10 µm thick) of a long-chain alcohol (LCA) with a known melting point temperature is used as a local temperature sensor: melting of the film indicates that the LCA melting point temperature was reached. Table 1 shows a set of LCAs with melting temperature ranging from 30 to 70 °C. The method for *in situ* temperature measurement based on melting of LCAs or other hydrocarbons with known melting point temperature was adopted from^16^.

**Table 1:** Melting temperatures of LCAs useful for CellEraser’s calibration (www.sigmaaldrich.com).

No	LCA name	Abbreviation	Melting point	Sigma-Aldrich product numbers
1.	1-Tridecanol	1-TD	31–34 °C	T57630
2.	1-Tetradecanol	1-TTD	38–39 °C	87158
3.	1-Pentadecanol	1-PD	41–44 °C	412228
4.	1-Hexadecanol	1-HXD	49–50 °C	258741
5.	1-Heptadecanol	1-HD	52–54 °C	241695
6.	1-Octadecanol	1-OD	58–59 °C	258768
7.	1-Nonadecanol	1-ND	60–61 °C	286842
8.	1-Docosanol	1-DS	65–72 °C	169102

The calibration procedure is performed under conditions identical to the conditions at which the cells would be treated: the same type of cultureware is used, the internal surface of its bottom is coated with the LCA film, and the same volume of the same culture medium is added (LCAs are practically insoluble in water^17^). The LCA film melts when the culture medium intercalated into or directly above the film is heated via application of the millimeter wave radiation. Dry film could not be melted by MMWs as absorption of millimeter wave radiation by LCA, higher alkanes or oils is negligible^13^.

**Figure 2:**
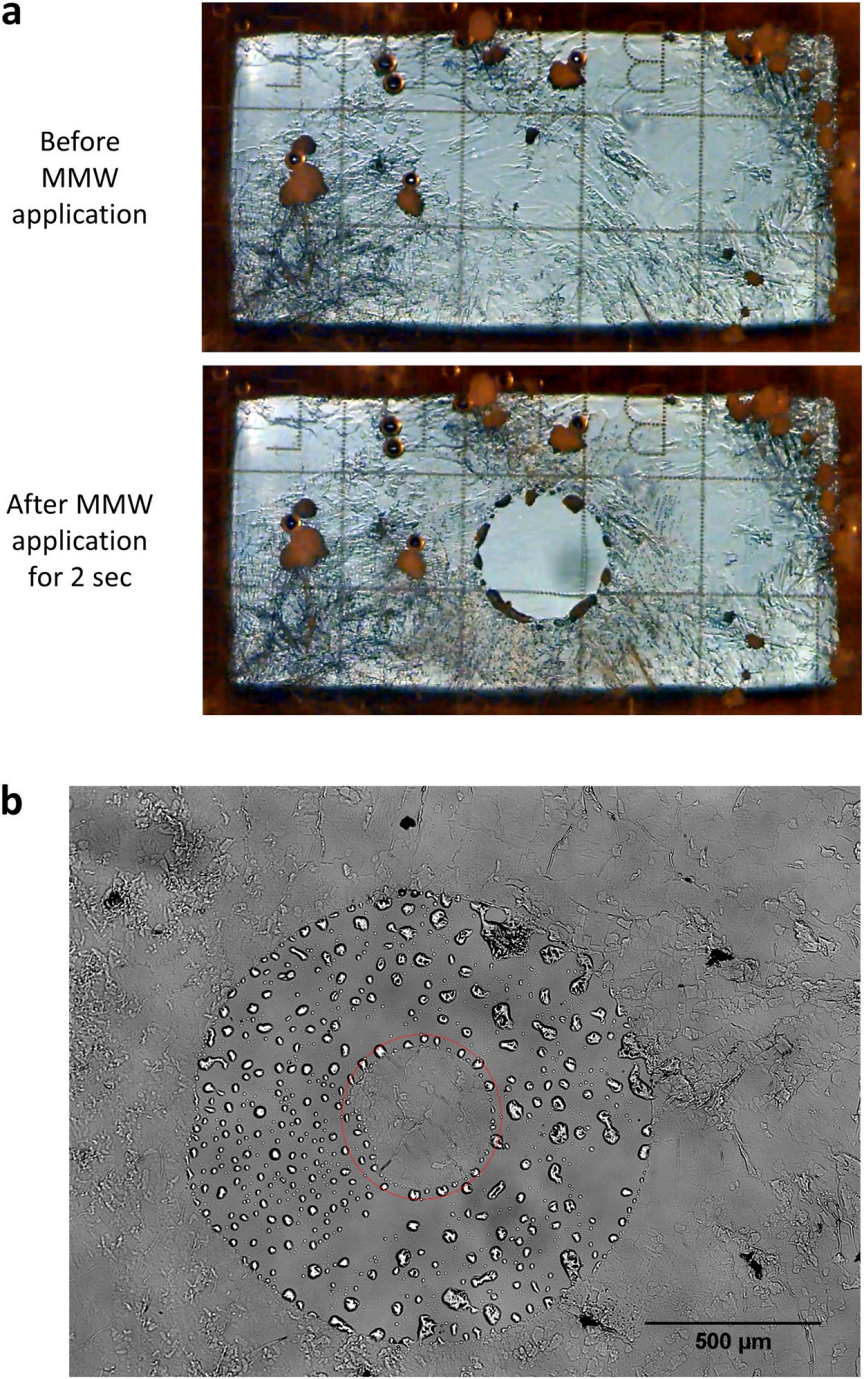
Calibration via LCA film melting. (**a**) Melting of an LCA film by MMW heating of the water above it. The film was formed from 1-Hexadecanol (melting point temperature 49–50 °C) on an ibidi chambered coverslip with 500 µm grid. The upper image was acquired before and the lower – after application of the millimeter wave radiation for about 2 seconds. The inner boundary of the end face of the output waveguide is seen on the perimeter of each image. The images were cropped to show only the area inside the waveguide. The upright microscope version of the instrument was used with a 4x objective (see also Supplementary Movie 2). (**b**) The ring melted in a 1-HXD film (see Supplementary Movie 5) using the inverted microscope version of the CellEraser instrument, 4x objective. The film was formed on a coverglass bottom 35 mm dish. The red circle indicates the size of the “eraser” − 500 µm. The diameter of the central unmelted patch was set to be 400 µm. Experiments were repeated at least 10 times, with similar results obtained each time; representative data from a single experiment are shown; refer to Fig. 3 for details on repeatability.

Upper image in Fig. 2a shows an unmelted 1-Hexadecanol (1-HXD) film formed on an ibidi chambered coverslip with an end face of the output waveguide positioned below it – the inner edge of the waveguide is seen on the perimeter of the image. 1-HXD was chosen as film material because, if heated above 1-HXD melting temperature, mammalian cells will not survive^18^. Application of the millimeter wave radiation for about 2 seconds melts an area 0.55 mm in diameter in the 1-HXD film (lower image in Fig. 2a). The melting process may be visualized with the upright microscope (Supplementary Movie 2). The inverted microscope version of the CellEraser allows imaging of the film only after the melting is finished (Supplementary Movie 3).

During the heating process, convection causes the heated medium to move upward and towards the center of the heated spot to replenish the volume moved upward (Fig. 1b, Supplementary Movie 1). The centripetal flow creates a sharp temperature gradient at the hot spot borderline judging by the well-defined boundaries of the melted area. This assures that the cells adjacent to the region of exposure will not be affected.

By adjusting exposure parameters such as the radiation power and exposure time of the millimeter wave radiation, and the vertical position of the output waveguide, a bigger or smaller melting area may be attained ranging from 0.2 to 1.5 mm in diameter for 94 GHz radiation. Thus, the equipment can be calibrated to treat cells in areas with different diameters.

The film could be moved horizontally to melt it at a different X-Y position. Repeating the process allows for melting of any desired pattern in the film. This may be done manually or with the use of a software-driven motorized microscope stage. For example the instrument could be programmed to melt a ring in the film - the same routine could be run on a cell culture to isolate a patch of desired cells. The ring melting was implemented via a script written in Micro-Manager^19^ by remotely on/off triggering of the MMW source and by driving the movement of the microscope stage. The process is shown in Supplementary Movie 4 for the upright microscope and in Supplementary Movie 5 for the inverted microscope. Figure 2b shows the end result of the ring melting in the Supplementary Movie 5. The size of the central unmelted patch could be adjusted and the ring width could be widened by increasing the number of concentric overlapping cycles of heating. The automation of the CellEraser could be implemented in any software for automated imaging (MethaMorph, NIS-Elements, etc.) that allows customization via macros, journals or other script writing capabilities.

**Figure 3:**
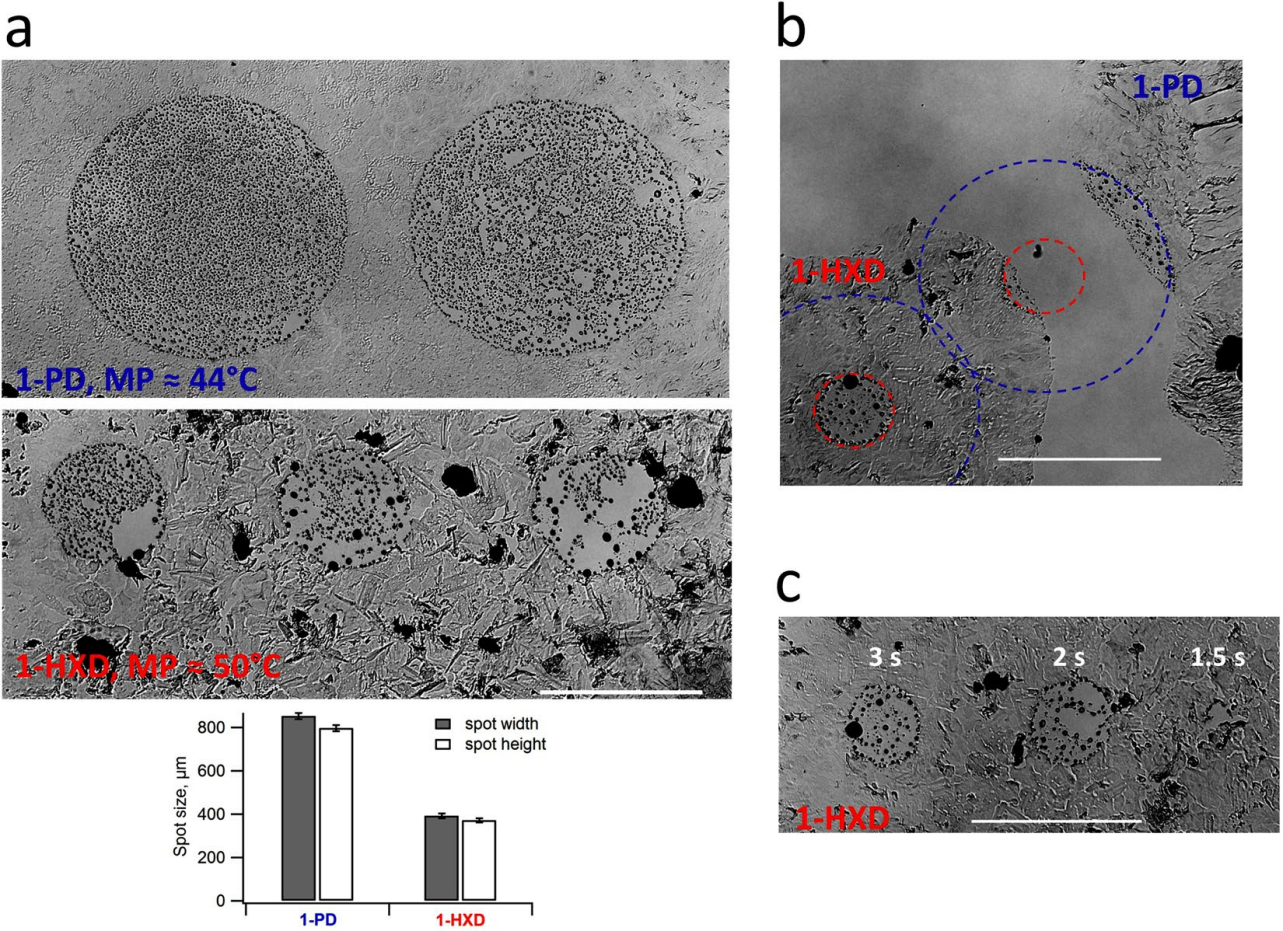
Characterization of the heated spot by LCA melting. Two LCA films were formed in different areas of a coverglass bottom 35 mm dish. The LCAs were 1-Pentadecanol (1-PD) with the melting point (MP) of about 44 °C, and 1-Hexadecanol (1-HXD) with MP of about 50 °C (see Table 1). The distance between the end face of the output waveguide and the bottom of the dish was 200 µm. About 2 ml of water was added to the dish. (**a**) Upper image: the millimeter wave radiation power was adjusted to melt a 850 µm spot in 1-PD film after 3 s exposure. To address the repeatability, multiple spots were melted (two are shown) by repeating the melting process in different X-Y positions without changing any other parameters. The spots were 850 × 800 µm ovals, not the perfect circles. Lower image: when the same melting was tried in the 1-HXD area, the spot size decreased to 390 × 370 µm (three spots are shown). The graph at the bottom shows the average spot width and height for both LCAs, error bars are the s.d. (n = 15). (**b**) The melting procedure was tried (with slightly lower MMW power compared to panel a) where the 1-PD and 1-HXD films were in close proximity to each other: parts of both films were melted simultaneously with the 1-PD melting spot area encircled by blue dash line and the 1-HXD one – by the red dash line. The melting was repeated in the 1-HXD area. (**c**) The effect of the MMW exposure time (indicated in white) on the size of the melting spot showing that at least 2 second exposure is required to achieve stable melting. The panel also demonstrates the minimal stable spot size of about 200 µm. The inverted microscope version of the CellEraser instrument was used with a 4x objective, the scale bar is 500 µm. The melting spot size varied with the s.d. ≤ 5% (n ≥ 10) assuming that the MMW power, the distance between the end face of the output waveguide and the bottom of the dish, and the exposure time were kept unchanged.

Figure 3 gives an idea about the temperature distribution at the CellEraser hot spot judging by the melting spot sizes of two LCAs with different melting temperatures. Melting a 500 µm spot in a LHA film will not melt the film made from the next higher MP LCA from Table 1. Figure 3 also characterizes the heated spot in terms of repeatability, minimal stable size and exposure time. The accuracy of spot X-Y positioning is determined by the microscope stage and is best demonstrated during the ring melting (Supplementary Movies 4 and 5, Fig. 2b). The spatial resolution of the CellEraser is determined by the minimal stable size of the heated spot - about 200 µm (Fig. 3) though the sharp spot boundary allows achieving the cell-size resolution: it is possible to irreversibly damage only one of two adjacent cells.

#### Cell isolation

**Figure 4:**
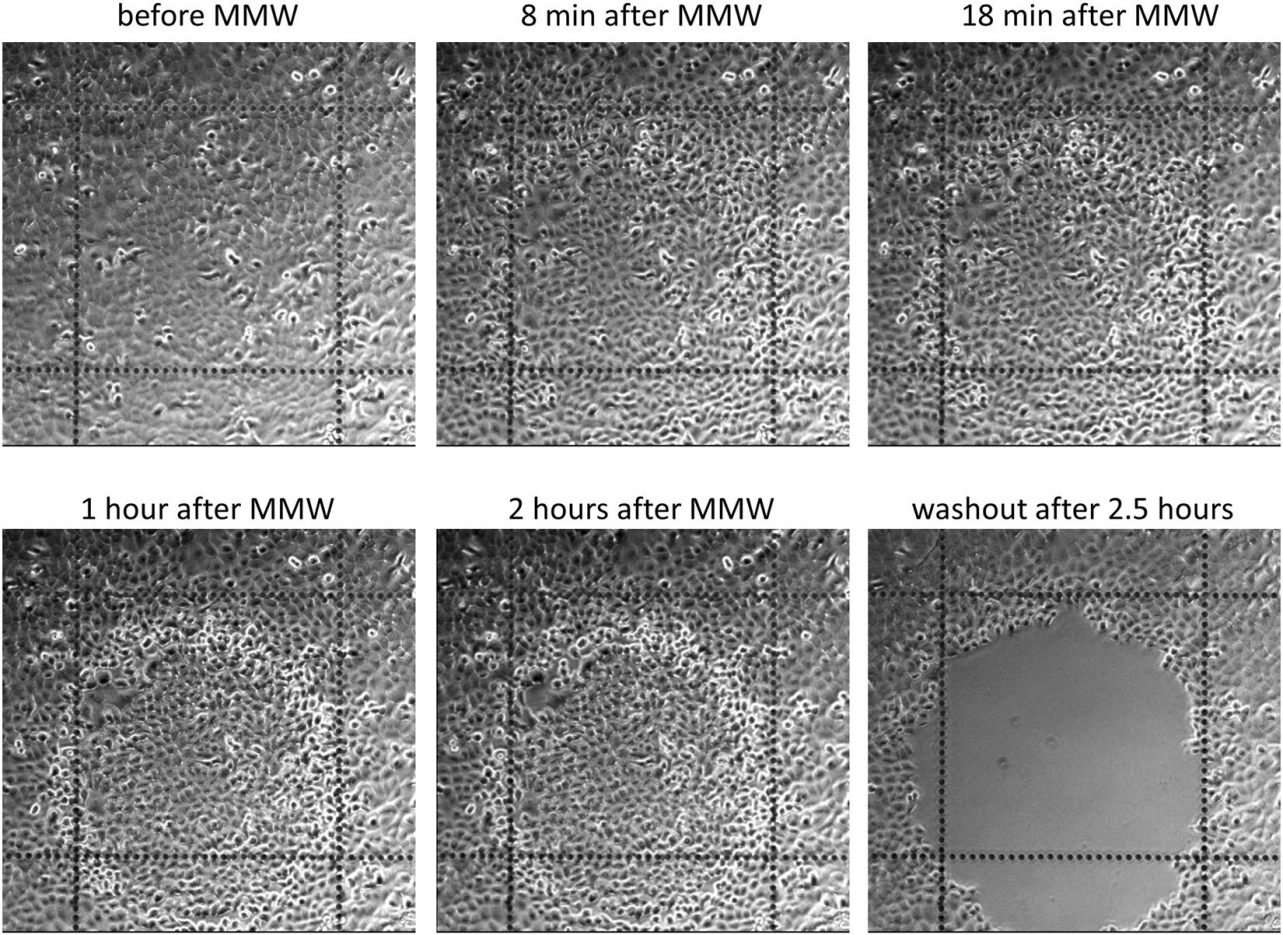
Spot “erasing” in adherent cell culture. A spot in a confluent A549 cell culture was heated to 50 °C by local application of MMW radiation for about 2 seconds. The images show the cell culture before and after the MMW treatment at indicated time points. The cells reacted to heating by rounding up and detaching from the substrate so that they can be removed by a media change (last image). Ibidi chambered coverslip with 500 µm grid, 10x objective (see also Supplementary Movie 6). Experiments were repeated at least 6 times, with similar results obtained each time; representative data from a single experiment are shown; refer to Supplementary Fig. 2 for details on repeatability.

After the calibration procedure is finished, the millimeter wave radiation is applied to an adherent cell culture to expose the unwanted cells to hyperthermic damage. The exposure parameters adjusted to obtain a desired diameter of the melting spot in the calibration procedure described above (Fig. 2a, Supplementary Movies 2 and 3) are used to damage a cell area of the same desired diameter. The results of the damage become visible 15–30 minutes after the treatment depending on the cell type. The cells exposed to lethal millimeter wave radiation power (i.e. heated above 50 °C) round up, detach from the substrate and can be washed out by a media change. Figure 4 and Supplementary Movie 6 illustrate the process by removing or “erasing” a 600 µm patch of unwanted cells from a confluent A549 cell culture leaving a clear (cell-free) surface surrounded by the confluent culture. See also Supplementary Fig. 2 for double spot erasing where the repeatability of the process is demonstrated.

**Figure 5:**
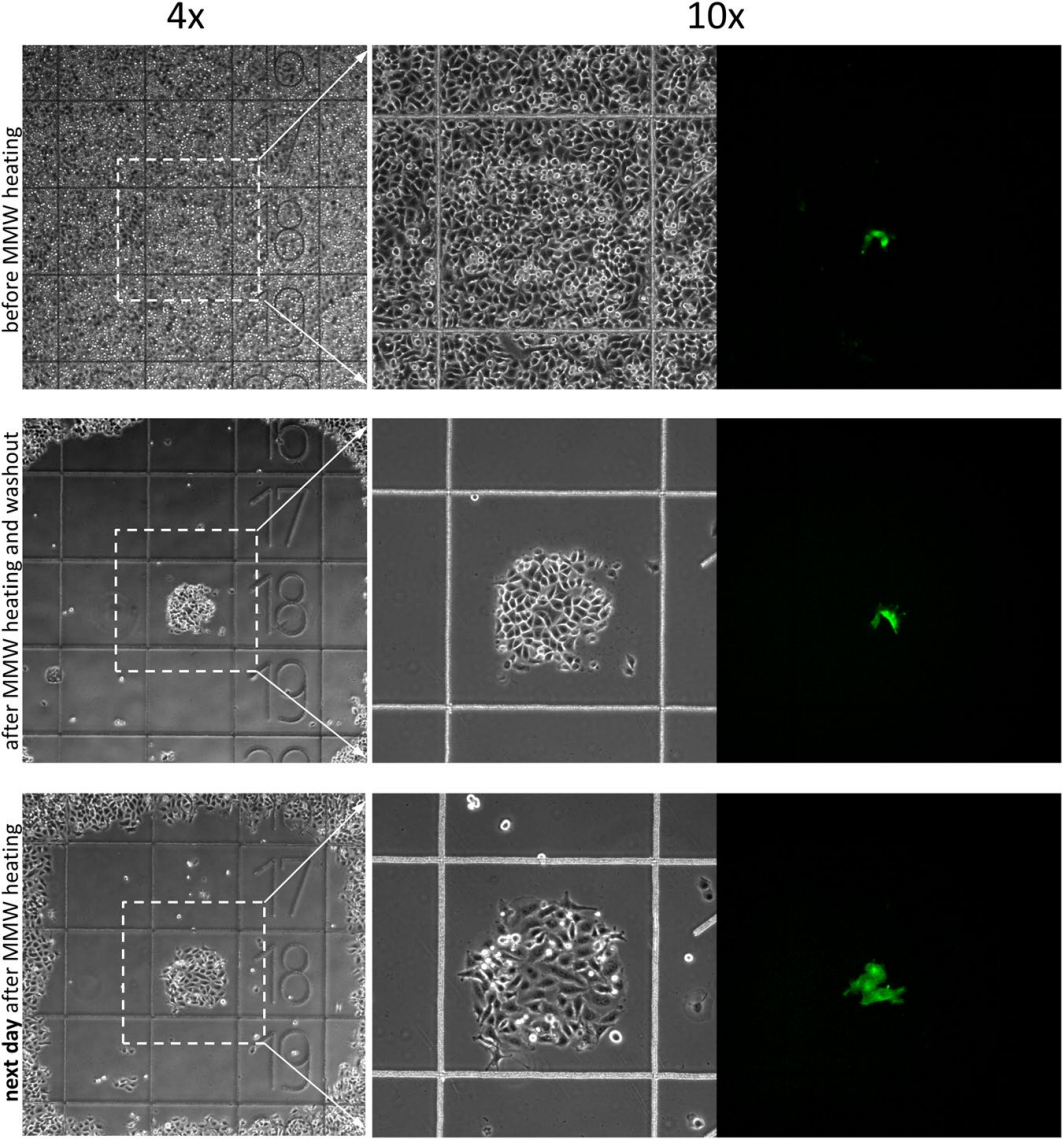
Isolation of GFP-expressing cells. (Upper row) Three GFP-expressing daughter cell were identified in a confluent A549 cell culture. (Middle row) Millimeter wave radiation was applied around a central patch of about 300 µm in diameter. The MMW application was done in three overlapping cycles with increasing radius. The culture was washed out 1 hour later clearing out the area around the central patch with fluorescent cells inside. The remaining single cells are floating cells from other areas of the culture that settled down after the washout. (Lower row) Next day observation revealed that now there were four fluorescent cells in the isolated patch. The survived border cells had migrated into the cleared area. Next rounds of “erasing” can enrich the fluorescent cells in the isolated patch and get rid of the cells on the periphery. Coverglass bottom 35 mm dish with etched 500 µm grid, 4x and 10x objectives, phase contrast and epi-fluorescence images. Experiments were repeated at least 4 times, with similar results obtained each time; representative data from a single experiment are shown.

Desired cells in the culture were isolated by removing cells around them using the automated procedure for ring melting in the LCA film described in the calibration section above (Fig. 2b, Supplementary Movies 4 and 5). First, the position of a region in the cell culture that contains the cells of interest had to be identified. For example, the desired cells were some rare fluorescent (GFP-expressing) cells (Fig. 5, upper row). Then, millimeter wave radiation was applied around the region containing the fluorescent cells leaving a central patch of a desired size untreated. The MMW mediated heating was done in overlapping cycles to increase the width of the heated ring of culture around the central patch. The damaged cells in the heated area were removed by a washout an hour later, exposing the central patch encircled by a cell-free ring of a desired thickness (Fig. 5, middle row). The border cells at the edge of the region of MMW exposure were not damaged. They continued to grow and actively migrated into the now empty area as seen by the next day observation (Fig. 5, lower row). The fluorescent cells inside the isolated patch were dividing. They could be further enriched inside the patch by more rounds of “erasing” aiming to isolate a cell line containing only the GFP-expressing cells. The cells surrounding the isolated patch could be pushed away from the patch or be completely eliminated.

A number of patches containing the desired cells can be isolated in the same cultureware. Single-cell cloning is also possible if the cells are plated in low enough density to allow colony formation – then all the unwanted colonies can be removed.

#### Creating the gap for wound healing assays

The wound healing assay is a standard *in vitro* technique for assessing collective cell migration in 2D^20^. This method mimics cell migration during wound healing *in vivo*. It requires creation of a cell-free area in a confluent monolayer inducing the cells to migrate into the gap to measure the rate of gap closure. Spot removal from a confluent cell culture by local MMW mediated heating (Fig. 4 and Supplementary Movie 6) resulted in a circular cell-free area or “wound” in the culture with well-defined boundaries. This wound or gap creation was reproducible, did not mechanically or chemically disturb the surrounding cells, and could be used in conventional wound healing assays instead of the current standards like scratching or inserts. The extra-cellular matrix (ECM) proteins most likely stayed intact in the gap surface after the cell detachment providing more physiological conditions for the cell migration. Supplementary Movie 7 shows a closure of a gap that was created by MMW application to a confluent cell monolayer. This novel gap-creation method to simulate wounding is convenient, non-contact and reproducible. It could be easily automated to adapt for microplate format to be used in medium to high throughput applications.

### Cells exposed to 50 °C have “leaky” nuclei

**Figure 6:**
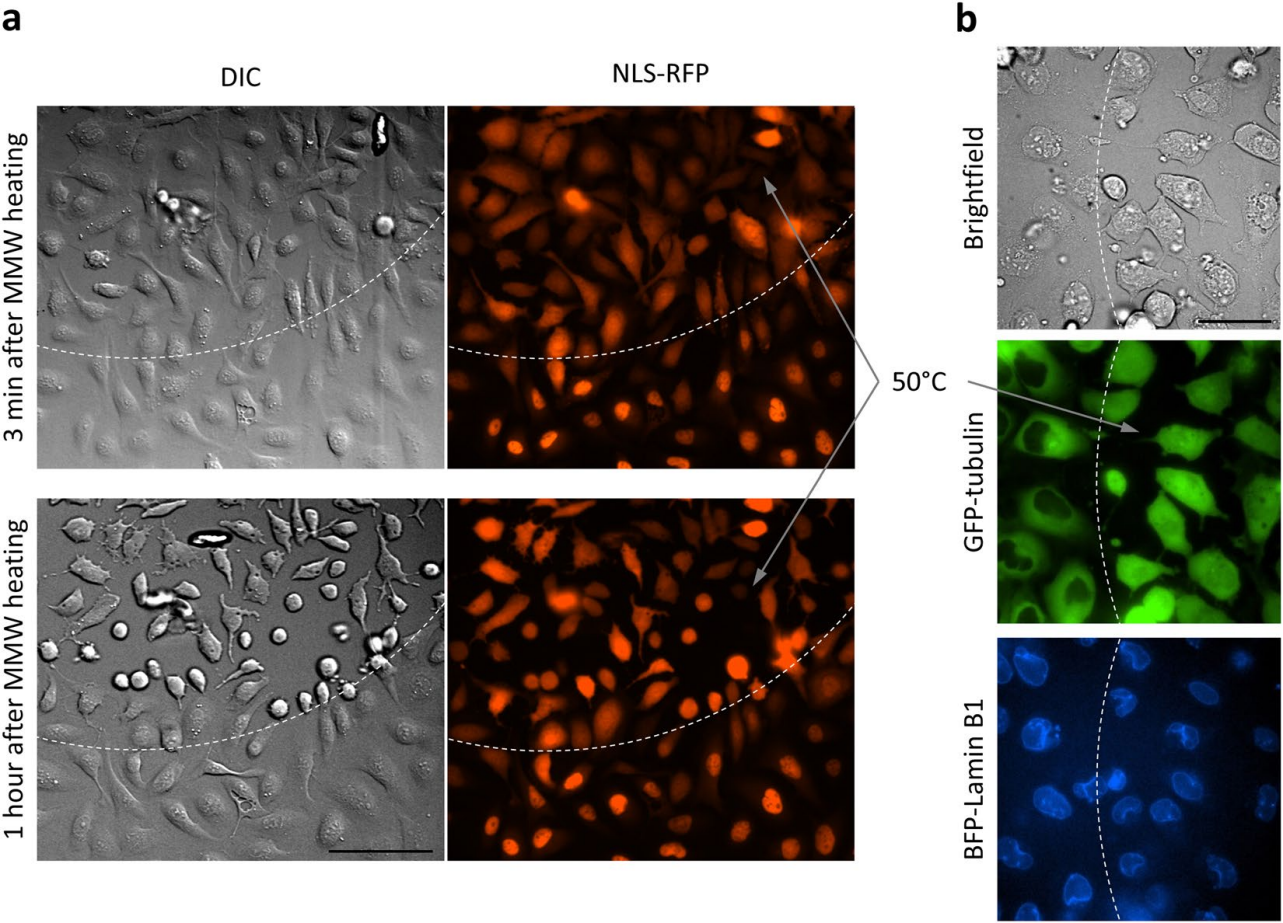
Nuclear “leakage” after 50 °C exposure. U2OS cells exposed to 50 °C for 2–3 seconds lost their nuclear envelope selectivity. Borders between MMW treated and untreated areas are shown as dashed lines. (**a**) In cells expressing NLS-RFP, heating induced the exit of NLS-RFP from the nuclei. 20x objective, the scale bar is 100 µm. (**b**) In cells expressing GFP-tubulin and BFP-lamin B1, heating induced the entry of GFP-tubulin into the nuclei while BFP-lamin distribution remained unchanged. 40x objective, the scale bar is 50 µm. Experiments were repeated at least 5 times, with similar results obtained each time; representative data from a single experiment are shown.

We used MMW radiation to heat an area of adherent cell culture to various temperatures for a few seconds and then observe changes in cell morphology or in distribution of fluorescent reporter molecules. Our main observation was that in cells exposed to 50 °C for 2–3 seconds, the nuclear envelopes (NEs) abruptly lost the selectivity in their permeability, i.e. the cell nuclei became leaky. We used fluorescent proteins (FP) fused to a nuclear localization sequence (NLS) as reporters to detect the loss of NE selectivity similarly to previously published work^21–23^. NLS-FPs normally had predominantly nuclear localization and rapidly escaped into the cytoplasm when NE integrity was lost. If cells expressing NLS-RFP were heated to 50 °C for 2–3 seconds, NLS-RFP irreversibly left their nuclei within seconds after heating and became evenly distributed between the nucleus and the cytoplasm (Fig. 6a, Supplementary Fig. 3a). On the other hand, there are other FP fusion proteins like GFP-tubulin that lack the NLS sequence and reside in the cytoplasm as they are not able to go through the nuclear pore due to their size. GFP-tubulin that normally never enters the nucleus, equilibrated between the nuclear and cytoplasmic compartments after the heating (Fig. 6b). Supplementary Figure 4 shows the kinetics of GFP-tubulin nuclear entry after MMW heating of cells to 50 °C.

Nuclear proteins that bind to DNA or have affinity to other nuclear components maintained nuclear localization even after the heat-induced disruption of NE selectivity. For example, BFP-Lamin B1, a polymerized NE protein (Fig. 6b) or GFP-53BP1, a protein involved in DNA repair^24^, remain nuclear even after RFP-NLS equilibrates between the nuclear and cytoplasmic compartments in the heated area (Supplementary Fig. 5a).

### No membrane rupture or protein denaturation after brief 50 °C exposure

**Figure 7:**
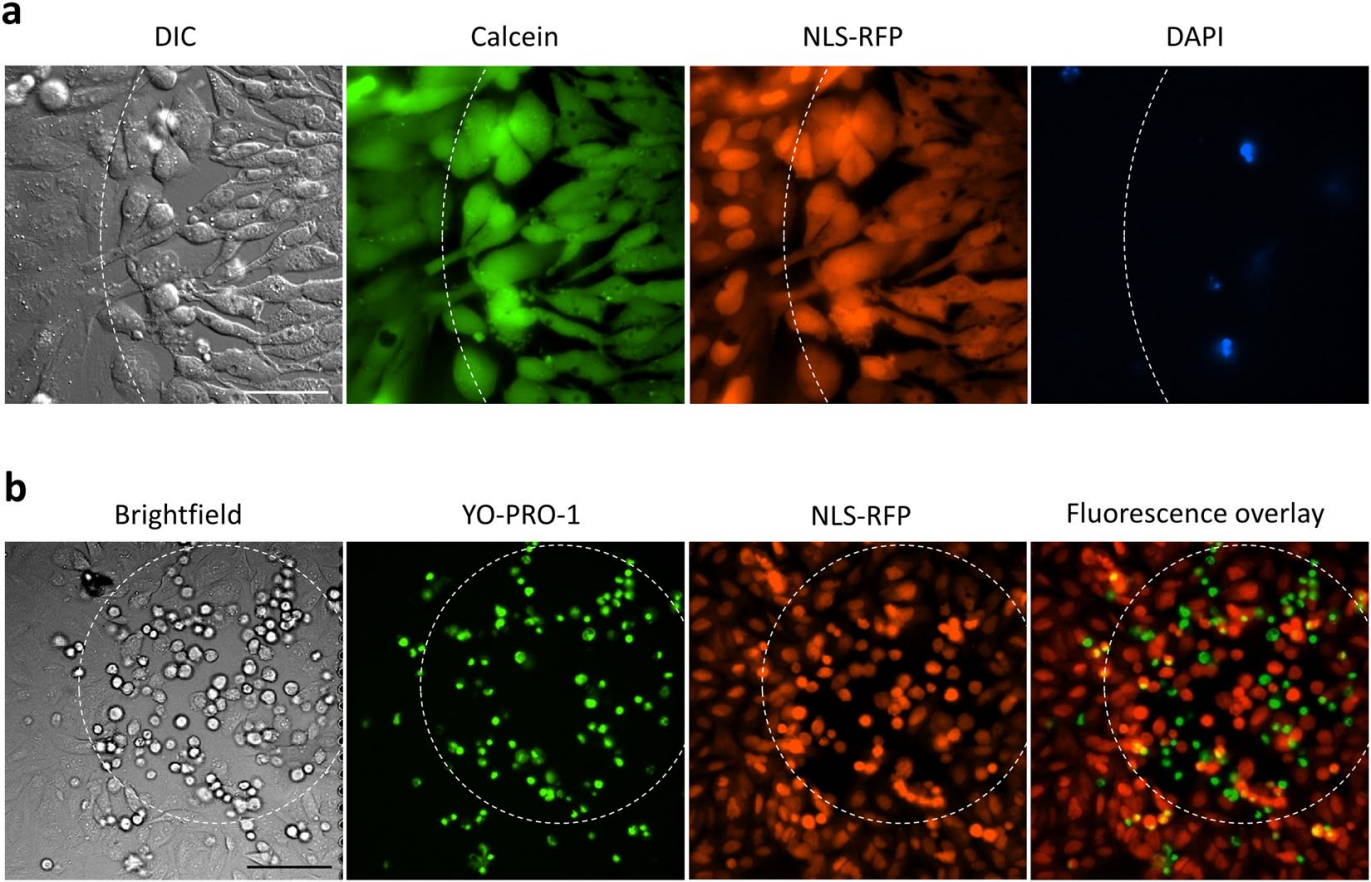
Cell viability assayed after 50 °C exposure. A spot in a confluent culture of U2OS cells expressing NLS-RFP was heated to 50 °C by local application of MMW radiation for 2–3 seconds. (**a**) Borderline (dashed) between MMW treated (right) and untreated (left) cells. 1 µg/ml Calcein-AM and 0.5 µg/ml DAPI were added after heating and images were acquired 30 minutes later. Cells with leaky nuclei and cells with intact nuclear selectivity were separated at the borderline as seen from NLS-RFP distribution. All cells were capable of cutting off the AM ester groups and retaining freed Calcein as well as preventing DAPI from entering (a few DAPI stained cells were most probably dead before MMW application). 40x objective, the scale bar is 50 µm. (**b**) The area within the dashed circle was MMW treated and the cells were incubated under environmentally controlled conditions for about 17 hours (see Supplementary Movie 8). Then an apoptotic marker YO-PRO-1 (1 µM) was added and the cells were imaged 30 minutes later. Cells in the heated area were rounded and detached, some of them have floated outside the heated area. Some cells surrounding the heated area have migrated inside it and did not lose their nuclear selectivity. Some of the detached cells became YO-PRO-1 positive and also NLS-RFP negative as there are only a few yellow cells on the overlay. 20x objective, the scale bar is 100 µm. Experiments were repeated at least 3 times, with similar results obtained each time; representative data from a single experiment are shown.

Cells with “leaky” nuclei (due to exposure to 50 °C for 2–3 seconds) started to round up and to lose their anchorage to the substrate, and finally detached from it within 1–2 hours (Supplementary Fig. 3b, Movie 6) but the content of those cells (proteins, DNA, etc.) did not leak out to the culture medium. Application of Calcein-AM to cells briefly heated to 50 °C revealed that these cells had normal esterase activity i.e. they were able to convert non-fluorescent Calcein-AM into fluorescent Calcein. So the rounding cells that lost the NE selectivity were Calcein positive. Fluorescent proteins could not escape the heated cells and a cell-impermeable DNA stain DAPI could not enter them suggesting that their plasma membranes were intact (Fig. 7a). The integral intensity of the FP signal per cell did not drop indicating that brief exposure to 50 °C does not induce denaturation of cellular proteins (Fig. 6, Supplementary Fig. 3a).

### “The temperature of no return”

It was at least several hours after exposure to 50 °C for 2–3 seconds when the cells started to show signs of degradation: they turned apoptotic as demonstrated by labeling with an apoptotic marker YO-PRO-1^25,26^. Figure 7b shows that some of the rounded and detached cells became YO-PRO-1 positive 17 hours after the heating (Supplementary Movie 8). Most of these cells were also NLS-RFP negative indicating that their plasma membranes were compromised. Note that the cells surrounding the heated area migrated inside it and did not lose their nuclear selectivity (Fig. 7b). All of the heated and now detached cells were destined to die as they never reattached back to the substrate and were losing their membrane integrity.

According to survival data of mammalian cells at elevated temperatures, it takes minutes to kill the cells at 48 °C^18,27^ (Supplementary Fig. 6). Higher temperatures (about 50 °C) should sharply shorten the survival time to seconds. However, technically it is very difficult to heat the cells to 50 °C only for a few seconds and then return them back to the physiological temperature. Local heating by MMW radiation allows for achieving that.

Our data indicate that mammalian cells briefly exposed to 50 °C at first did not show obvious signs of damage other than the loss of nuclear selectivity. Changes in morphology started to occur minutes later but the plasma membrane stayed intact for hours. The NE integrity was never restored in the heated cells. The damage to NE barrier function always induced cell detachment from the substrate (partial or complete). These cells never divided and eventually died through apoptosis. The effect was reproducible in a number of immortal and stem cell lines (data not shown).

That is why we call 50 °C “the temperature of no return” as a cell will never recover even after a short exposure to it. Lower temperatures require longer exposure time to trigger the irreversible damage, and the earliest indicator of that damage is the loss of nuclear selectivity (data not shown). At 42 °C mammalian cells can survive long term^18,27^ (Supplementary Fig. 6).

### Membrane rupture without protein denaturation after brief 60 °C exposure

**Figure 8:**
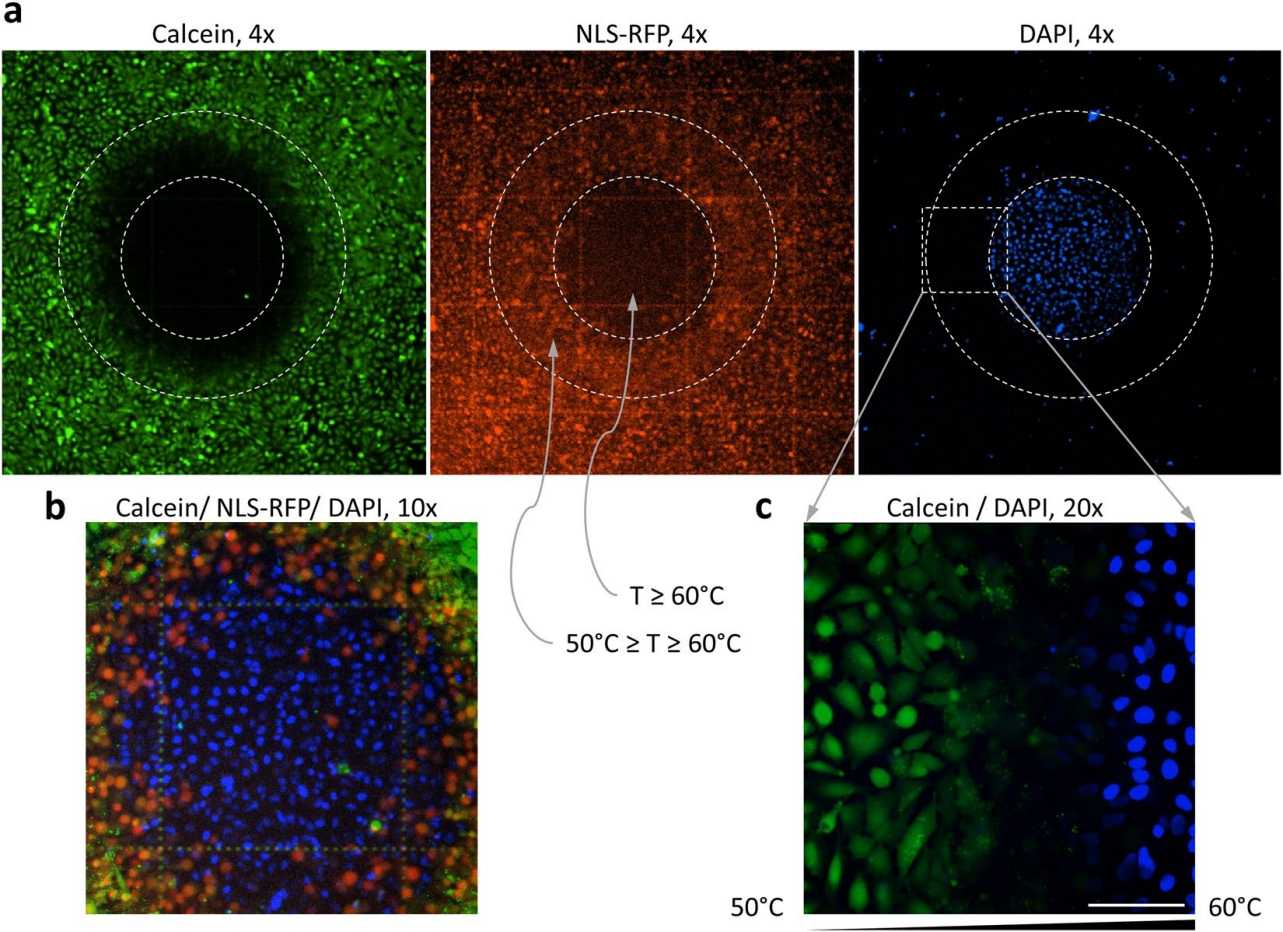
Cell viability assayed after 60 °C exposure. A spot in a confluent culture of U2OS cells expressing NLS-RFP was heated to 60 °C by local application of MMW radiation for 2–3 seconds. 1 µg/ml Calcein-AM and 0.5 µg/ml DAPI were added after heating and the cells were imaged 30 minutes later. (**a**) Areas where the temperature (T) reached 60 °C and gradually dropped from 60 °C to 50 °C are shown by the dashed circles for all three viability markers. The sizes of 50 °C and 60 °C dashed lines were determined by melting two LCA films formed in close proximity to each other. The LCAs were 1-Hexadecanol (1-HXD, MP = 50 °C) and 1-Octadecanol (1-OD, MP = 59 °C) - see Table 1. NLS-RFP channel serves as another (indirect) indicator of the temperature: RFP starts to leak out of the nuclei at 50 °C and out of the cell at 60 °C, 4x objective. (**b**) Zoomed-in image of the heated center spot in A overlaying all three markers, 10x objective. (**c**) Further zoomed-in image of the transition area at the edge of the heated spot (dashed square) where the temperature gradually dropped from 60 °C to 50 °C. The image is an overlay of Calcein and DAPI channels, 20x objective. Ibidi chambered coverslip with 500 µm grid, the scale bar is 100 µm. Experiments were repeated at least 3 times, with similar results obtained each time; representative data from a single experiment are shown.

Rising temperatures was shown to change cell membrane fluidity and permeability^28^. Figure 8 illustrates NLS-RFP expressing cells that were first heated to 60 °C for 2–3 seconds and then Calcein-AM and DAPI were added to the culture medium to probe for cell viability. The images indicate that a short heating to 60 °C resulted in an abrupt loss of the plasma membrane integrity: the cells became Calcein negative, NLS-RFP leaked out of them and a cell-impermeable DNA dye DAPI was able to enter the cells simultaneously with the NLS-RFP leakage. So the heated region was DAPI positive and RFP/Calcein negative. It also was YO-PRO-1 positive (Supplementary Fig. 7). We noticed a narrow region that was RFP positive but Calcein and DAPI negative – it was reflected in a bigger size of the dark spot in Calcein channel compared to smaller RFP negative and DAPI positive spots (Fig. 8a). Cells in this region had a non-leaky plasma membrane that prevented RFP-NLS leakage and DAPI entering so Calcein could not leak out, yet there was no Calcein signal (Fig. 8b,c). The absence of Calcein-AM to Calcein conversion suggests that esterase enzymatic activity was significantly attenuated. Thus local heating of a cell culture to 60 °C for 2–3 seconds resulted in two damaged areas: 1) the center spot where cells were dead i.e. DAPI positive and RFP/Calcein negative; 2) a zone of cells with leaky nuclei around the center spot - the temperature in this zone gradually dropped from 60 °C to 50 °C and the cells were RFP positive, DAPI negative and mostly Calcein positive. The leaky nuclei area included a narrow intermediate zone (where the temperature was slightly lower than 60 °C) with Calcein negative cells.

However, DNA bound, cytoskeletal or other FP fusion proteins that were not free-floating inside the cell and could not leak out of it, were still fluorescent after brief exposure to 60 °C that caused membrane rupture and leakage of cytosolic proteins. For example, GFP-53BP1 did not lose its fluorescence and was localized in the nuclei while RFP leaked out (Supplementary Fig. 5b). FP fusions with polymerized proteins like actin, tubulin or lamin also maintained fluorescence and its pattern after brief heating to 60 °C (data not shown) suggesting that there was no significant protein denaturation.

The number of DNA double-strand breaks (DSBs) in a cell can be detected by monitoring GFP-53BP1 distribution pattern because as a part of the DNA repair machinery this protein forms bright foci at the sites of DSBs^24^. In cells heated to 60 °C for 2–3 seconds and in unheated cells the number of GFP-53BP1 foci stayed about the same (Supplementary Fig. 5b). In control experiments we used Etoposide to chemically induce DSBs in order to verify the GFP-53BP1 functionality. We observed multiple bright foci in Etoposide treated cells (Supplementary Fig. 5c). Our data indicate that brief exposure to 60 °C did not induce DNA DSBs.

### “Thermofixation”

**Figure 9:**
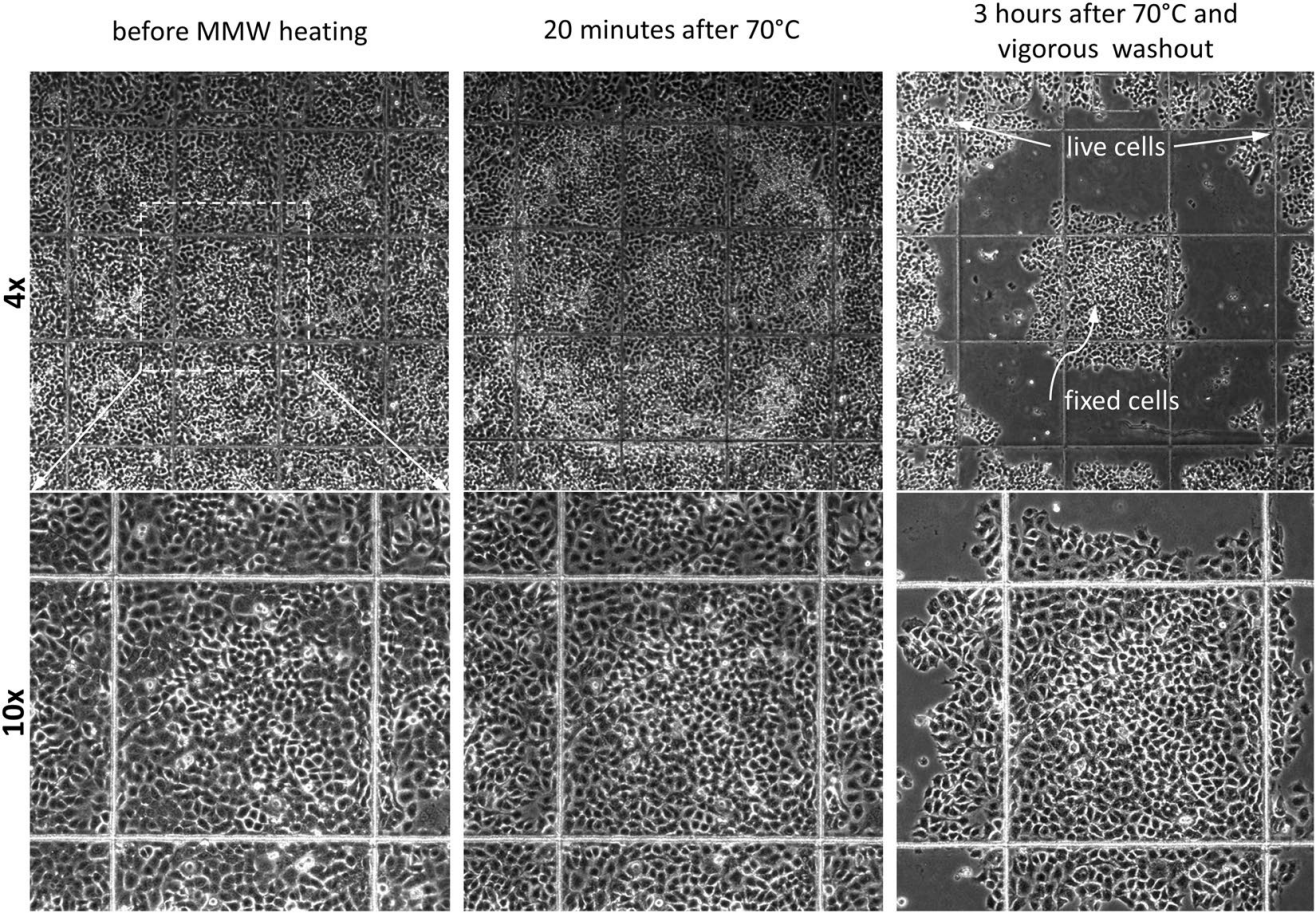
“Thermofixation”. A spot in a confluent A549 cell culture was “thermofixed” by heating to 70 °C using local application of MMW radiation for 2–3 seconds. The images show the cell culture before and after the MMW treatment at indicated time points. Cells in the center region exposed to 70 °C remained tightly attached to the substrate even after a vigorous washout (right column) while cells in the border region where the temperature gradually dropped from 70 °C to 50 °C, detached from the surface and were washed away. Coverglass bottom 35 mm dish with etched 500 µm grid, 4x and 10x objectives. Experiments were repeated at least 3 times, with similar results obtained each time; representative data from a single experiment are shown.

**Figure 10:**
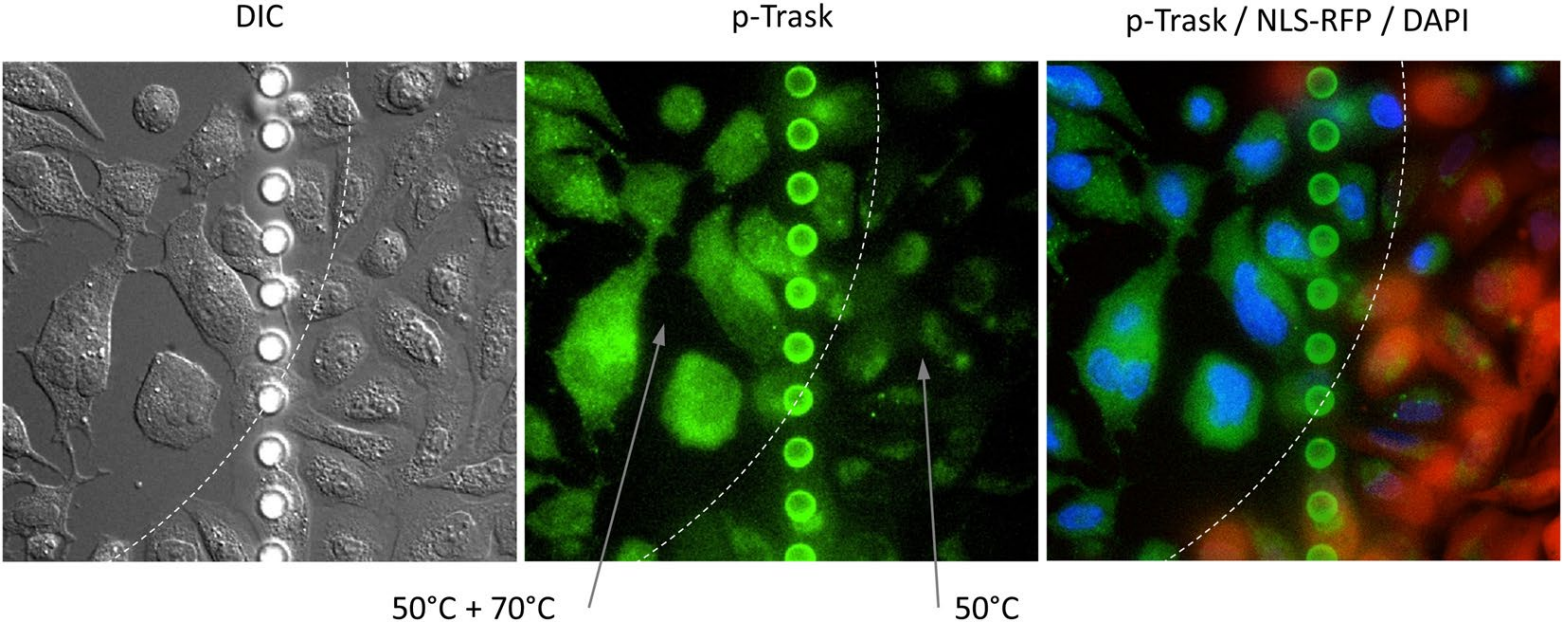
Heat-induced Trask phosphorylation. U2OS cells expressing NLS-RFP were heated to 50 °C for 2–3 seconds using MMWs. This led to cell rounding and partial detachment from the substrate within 30 minutes. Then an area within the rounded cell culture (to the left of the dashed line) was “thermofixed” by heating to 70 °C for 2–3 seconds in order to denature the proteins and permeabilize the membrane. Finally the cells were blocked, p-Trask (pY743) primary and secondary antibodies were applied, and 0.5 µg/ml DAPI was added. The images show the edge of the “thermofixed” area. The right image is an overlay of all three fluorescence channels. 40x objective, Ibidi chambered coverslip with 500 µm grid, the dots of the grid line were made at every 20 µm. Experiments were repeated twice with similar results obtained.

Figure 9 shows how adherent cells reacted to a MMW induced short-term heating to 70 °C. Cells in the center region exposed to 70 °C remained tightly attached to the substrate even after a vigorous washout while cells in the border region where the temperature gradually dropped from 70 °C to 50 °C, detached from the surface and were washed away. The cells in the central patch looked fixed as they became resistant to washing and stayed attached for days without any noticeable changes in cell morphology. We called this phenomena “thermofixation” by analogy to the conventional chemical fixation. We hypothesize that the temperature jump to 70 °C led to a quick denaturation of cellular proteins in addition to the cell membrane permeabilization observed after 60 °C exposure. This was supported by the fact that FP fusion proteins like GFP-53BP1 or GFP-Lamin B1, not capable of leaking out of the cell, lost their fluorescence after heating to 70 °C for 2–3 seconds (data not shown). “Thermofixed” cells could be used for immunohistochemistry or immunofluorescence and do not require the chemical fixation and permeabilization steps (Fig. 10).

### Heat-induced Trask phosphorylation

We noticed that the heat-induced cell rounding and detachment from a substrate is similar to the detachment of adherent cells during mitosis – a process during which adherent cells must rapidly sever cell–surface and cell-cell engagements to execute mitotic functions. It was reported that a transmembrane glycoprotein Trask (CDCP1) is tyrosine phosphorylated by src kinases during the cell-detachment phase of mitosis. The phosphorylation of Trask was tightly linked to cell retraction from the substrate and associated with anchorage loss in epithelial cells^29,30^. When cells were induced to detach by trypsin or EDTA, Trask phosphorylation started to occur at the onset of cell rounding before detachment, peaked when cells were completely detached and was maintained for prolonged periods as long as cells were kept in suspension^29^. If Trask phosphorylation is forcibly maintained by overexpression, it leads to the loss of cell adhesion and a detached phenotype^30^. We hypothesized that the heat-induced nuclear leakage followed by the cell rounding and detachment may also include Trask tyrosine phosphorylation consistent with its role in the negative regulation of cell adhesion.

Published methods of phosphorylated Trask (p-Trask) detection were based on custom p-Trask antibodies that were made by immunizing rabbits with a phospho-peptide immunogens containing sequences centered around phosphorylated tyrosine 734^31,32^ or 743^29,33^ of Trask. The only commercially available p-Trask antibodies (monoclonal pY734-Trask and pY743-Trask) were developed for western blot applications that require denaturing of the protein sample by heating it to 95–100 °C for 5 min to expose the epitope. We tried to immunostain p-Trask in trypsin-induced or heat-induced rounded/detached cells using pY743-Trask western blot antibody after a standard paraformaldehyde fixation and Triton X-100 permeabilization but could not detect any signal (data not shown). Next we decided to repeat the experiment using “thermofixation” instead of the chemical fixation to denature p-Trask and make the pY743 epitope sequence accessible to pY743-Trask antibody.

Figure 10 shows NLS-RFP expressing cells that were first briefly heated to 50 °C to induce cell rounding and partial detachment from the substrate. Then a part of the rounded cells (to the left of the dashed line in Fig. 10) was “thermofixed” by heating to 70 °C in order to denature the proteins and permeabilize the membrane. Finally the cells were blocked, primary and secondary antibodies were applied, and DAPI was added. p-Trask antibodies mostly bound “thermofixed” cells that become completely RFP-NLS negative and DAPI positive. The neighboring cells (to the right of the dashed line in Fig. 10) that were not heated to 70 °C were not permeabilized, still contained RFP-NLS and had no p-Trask staining.

Our data confirmed Trask phosphorylation in cells heated to 50 °C for 2–3 seconds and suggest that Trask plays an important role in cell retraction, rounding and loss of anchorage in response to heat. We also demonstrated that “thermofixation” enables the use of western blot antibodies for immunofluorescence.

## Discussion

### MMW induced heating and its applications

We have developed a novel and unique cell manipulation technology that uses millimeter wave radiation for local heating of adherent cell cultures. It allows elimination of unwanted cells in the cultures by brief local heating to 50 °C that leads to selective hyperthermic damage first seen as cell detachment. The technology is implemented as a device, CellEraser - a compact microscope attachment that can remove or “erase” the unwanted cells from an adherent cell culture in an automated manner.

CellEraser’s key features:

- Allows *in situ* elimination of unwanted cells in adherent cell culture while it is propagating
- Controls the temperature and the size of the heated spot
- Does not heat the bulk culture medium or the cultureware material
- Non-invasive: does not compromise culture sterility, does not require addition of chemicals
- Does not affect the desired cells (no shear stress), does not induce immediate lysis of unwanted cells
- Works consistently among different mammalian cell types
- Can be used for cells grown in standard cultureware
- Inexpensive alternative to other cell isolation systems (LMD, CellCelector^34^, etc.)
- Safe for the user

There are no other commercially available systems/techniques that can non-invasively eliminate unwanted cells from adherent cell cultures. Current cell isolation and cell cloning approaches are based on flow sorting (FACS - fluorescence activated cell sorting), laser microdissection^10^, micromanipulation (micropipette transfer^35^ or articulated arm retrieval^34,36^), limiting dilution^37^, and microfluidics^38^. All of these techniques have limitations especially when applied to extremely rare or fragile cells or cells that exhibit low efficiency of outgrowth from a single-sorted cell. *In situ* cell isolation by CellEraser could overcome these issues as the desired cells are not affected in any way during the “erasing” process. The unwanted cells could be temporary left “unerased” on the periphery of the culture, away from the desired cells, to constantly condition the media and help the desired cells reach densities high enough when it is safe to eliminate all of the unwanted cells.

Possible applications of the instrument:

- Purification of induced pluripotent stem (iPS) cells and embryonic stem (ES) cells cultures from differentiating cells or isolation of differentiated cells
- Single-cell cloning by eliminating all unwanted colonies and leaving only one colony in a well
- Generation of tumor cell cultures originated from cancer patient tumor biopsies by removing the non-cancer cells to prevent them from taking over the culture
- If combined with a high content imager equipped with environmental control, the instrument can allow manipulation of the growing cultures in a high throughput mode
- A novel gap-creation method for wound healing assays
- Heating a culture area to sub-lethal temperatures (40–43 °C) for studies of thermo-activated cancer drugs, hyperthermia effects on cancer vs normal cells in culture, etc.

We also showed that CellEraser provides a simple and fast alternative to conventional chemical fixation - “thermofixation”, a short-term MMW heating of a small area in a live cell culture to 70 °C (Fig. 9). Conventional immunostaining of “thermofixed” cells could be performed without the permeabilization step for intracellular targets and western blot antibodies could be used as “thermofixation” denatures cellular proteins (Fig. 10). Different spots of a cell culture in a well could be “thermofixed” at different time points creating “time stamps” to record what is happening to the cells over time. Such “time stamping” followed by immunostaining could reveal the dynamics of a signaling pathway that would be recorded in a single well.

We are planning to facilitate the cell “erasing” by treating the cell culture with MMW radiation at the presence of cold (room temperature) trypsin or other digestive enzymes whose activity is low at 20–22 °C. MMW heating a spot in the cell culture to 38–40 °C should result in a constant supply of active (hot) trypsin to the spot due to the convection flow. This will accelerate cell detachment in the heated area and should enable observing the “erasing” in real time, maybe within tens of seconds. The detached cells will not be killed in the process. They can be collected, analyzed (if necessary) and propagated. In addition to *in situ* cell isolation, such technique could be used for local temperature-activated enzymatic sampling of desired cells (e.g. genotyping of multiple single-cell colonies grown in the same well/dish).

### Leakage of the nuclear envelope

We used MMW radiation to study the effect of quick and brief heating on mammalian cells. To control the temperature we applied a simple and reliable method of thermometry based on melting of LCA films formed on the cultureware bottom: melting of the film indicated that the LCA melting point temperature was reached (Fig. 2).

We demonstrated that nuclei of the cells heated to 50 °C for 2–3 seconds became “leaky” (Fig. 6) though the signal of the viability dyes (calcein, DAPI, and YO-PRO-1) indicated that the cells were alive for hours after heating: they had normal intracellular esterase activity and plasma membrane integrity (Fig. 7a). However, the heated cells did not survive long-term: they were not able to repair the loss in nuclear envelope selectivity, lost the ability to attach to the substrate and finally died via apoptosis (Fig. 7b).

It was reported that mammalian cells can survive temperatures of about 65 °C with exposure time not exceeding 5 seconds^39^. The authors relied on standard live-dead assays that indicate the enzymatic activity and/or the plasma membrane integrity, and the viability of the cells was assessed shortly after the heat exposure. The problem is that the heat effects could result in a delayed cellular damage and could take hours to develop. So the cell viability should be checked next day to judge about the effects of elevated temperatures.

Our main observation was that the cells were unable to recover after a thermal stress if such stress led to a loss in nuclear envelope selectivity – the earliest indicator of irreversible cell damage. To our knowledge, this finding was never reported before. The heat-induced NE leakage was observed as a loss in compartmentalization of intracellular proteins causing mislocalization of both nuclear and cytoplasmic protein components (Fig. 6) within two minutes after heating to 50 °C (Supplementary Fig. 4).

The main NE component is the nuclear double membrane that encapsulates the nuclear genome, regulates all nuclear trafficking of RNAs and proteins and prevents the passive diffusion of macromolecules between the nucleoplasm and the cytoplasm. Though we did not investigate the mechanisms of the NE selectivity loss, there are at least two possibilities: 1) rupture of the NE itself (the nuclear membrane and/or the lamina – the internal NE lining); 2) loss of the nuclear pore complex (NPC) selectivity.

The NE rupture was observed in cancer cells during cell migration through confining spaces^40^. Authors showed that NE of cells underwent repeated ruptures that allowed nuclear proteins to leak out and cytoplasmic proteins to leak in. Nuclear membrane protrusions (“blebs”) typically formed at sites where the nuclear lamina, particularly the lamin B1 network, was weak or absent. Depletion of the lamins A, C and B2 significantly increased the likelihood of NE rupture, which together with previous studies^21,22,41,42^ suggest that lamins are important for stabilizing the NE. Authors also showed that DNA double-strand breaks (detected as rapid formation of new RFP-53BP1 foci) coincided with the NE opening events.

Our observations of cells expressing FP-tagged lamin B1 (BFP-lamin B1) did not reveal any significant changes in BFP-lamin B1 organization in cells whose nuclei became “leaky” after heating to 50 °C compared to the neighboring unheated cells with non-leaky nuclei (Fig. 6b). Monitoring GFP-53BP1 distribution pattern after brief exposure to 50 °C (and even to 60 °C) showed no new foci formation (Supplementary Fig. 5) indicating that DNA double-strand breaks were not induced in the cells with compromised NE selectivity. Based on this data we thought that it is unlikely that the nuclear membrane and underlying lamin network was compromised by heating to 50 °C.

Then the hypothesis of heat-induced NPC damage looked more probable. It assumed that the mislocalization of nuclear and cytoplasmic proteins happened through “leaky” nuclear pores. It was reported that ageing-related deterioration of NPCs weakened the nuclear permeability barrier and lead to leakage of cytoplasmic proteins into the nuclear compartment^43^. Authors showed that NPC scaffold members did not turn over and had life-long residence at the NPC in non-dividing cells suggesting an idea that the presence of the same nucleoporins during the entire life span of post-mitotic cells comes at the physiological cost of ageing-related pore defects that lead to a loss of nuclear integrity^43^. A leakage of cytoplasmic proteins into the nucleus was also observed when NPCs were partially dismantled during mitosis^44^.

It was shown that mammalian cells could survive incubation at 43 °C for 1 hour but their conventional nuclear import was suppressed due to nuclear accumulation, nuclear retention, and recycling inhibition of importin α^45^. In our studies the cells were heated to higher temperatures that induced the irreversible loss of NE selectivity. We hypothesize that these temperatures triggered a rapid loss of the NPC functions that collapsed the macromolecular gradient across the nuclear membrane allowing the cytoplasmic proteins to enter the nuclear compartment and the nuclear proteins to exit into the cytoplasm. Since the biogenesis of the NPCs is restricted to the mitotic stages and ceased upon cell cycle exit^43^, the heated cells were not able to repair the leakage of the heat-damaged nuclear pores to restore the macromolecular gradient and self-terminated through apoptosis.

### Heat-induced cell detachment

We noticed a similarity between the heat-induced cell detachment and the cell-detachment that happens during prophase of mitosis in adherent cells. In both cases there is a nuclear envelope breakdown when the contents of the nucleus and the cytoplasm are mixed together. The NE disassembly precedes the detachment and most probably triggers Trask phosphorylation that also occurs in both heat-induced and mitotic detachment cases (Fig. 10 and^29^, respectively). The phosphorylation of Trask leads to an anchorage loss and a detached phenotype^29,30^. Although we did not study the mechanisms involved in heat-induced cell detachment, we hypothesize that initial cell reaction to heat have common features with mitotic detachment in terms of changes in cell physiology. However, the fate of heat-detached cells is quite different from dividing cells: they are not able to reattach back to the substrate, the lost NE selectivity never gets restored (possibly due to unrepairable thermal damage of the nuclear pores), and the cells eventually die through apoptosis (Fig. 7b).

Local heat-induced cell detachment can provide a useful means of taking a sample of an adherent cell culture without chemically or mechanically perturbing it - for example, culture sampling for the purpose of genotyping when splitting the cells is not desirable. A small area in the cell culture can be heated by MMW radiation to detach the cells in the area and the media can be changed an hour later to collect the cells for analysis. The unheated cells would not be affected in any way other than the media change. The process can be expanded to plate formats to sample clonal isolates in a high throughput mode.

## Conclusions

Our work is centered on a new and unique method of fast local heating applied to cell cultures. It uses millimeter wave radiation to achieve quick release of heat in a small surface volume. We discussed possible applications of this approach, as well as our findings that provide new insights into how a cell reacts to heat. We see the main application of the method as *in situ* cell isolation/culture manipulation via MMW-mediated irreversible hyperthermic damage. We also discovered that the loss of nuclear selective permeability serves as an early indicator of the irreversible cell damage that leads to cell detachment and apoptosis. “Thermofixation” is another application of the method we found that allows the use of western blot antibodies for immunofluorescence/immunohistochemistry. The instrument could be used for other applications including gap creation for wound healing assays. We hope CellEraser and the data obtained with its help will be useful for understanding the mechanisms of hyperthermia induced cell death and will be useful for enhancing or augmenting the outcome of cancer hyperthermia treatments.

## Methods

### Chemicals

Unless otherwise indicated, all reagents and materials used in this work were obtained from Sigma-Aldrich (Product Numbers are in parenthesis): DAPI (D9542), YO-PRO™−1 Iodide (Thermo Fisher Scientific, Y3603), Calcein AM (C1359), Etoposide (E1383). Description of long-chain alcohols (LCAs) used is summarized in Table 1. LCA films were formed from LCA solutions in 2-Propanol (190764).

### Cell lines and culture conditions

Human B lymphoblast RL (CRL-2261), lung carcinoma A549 (CCL-185), and osteosarcoma U2OS (HTB-96) cell lines were obtained from American Type Culture Collection (ATCC). Triple-tagged U2OS cell line (BFP-LMNB1/GFP-TUBA1B/RFP-ACTB, CLL1218) was obtained from Sigma-Aldrich. RL and A549 lines were grown in RPMI-1640 medium (R0883), U2OS lines – in McCoy′s 5 A medium (M8403. Both mediums were supplemented with 2 mM L-glutamine (G7513) and 10% fetal bovine serum (F2442). Cells were grown in 5% CO2 in air atmosphere, 37 °C. Trypsin-EDTA solution (T3924) was used for sub-culturing.

### Plasmids and transfection

BFP and TagBFP, GFP and TagGFP2, and RFP and TagRFP are synonymous for the fluorescent reporter genes/proteins in this manuscript. The BFP, GFP and RFP are monomeric fluorescent proteins originated from Evrogen and referred to as TagBFP, TagGFP2 and TagRFP respectively. Vectors pTagRFP-N (FP142) and pTagBFP-N (FP172) were obtained from Evrogen. NLS-RFP construct was made by inserting the NLS sequence (APKKKRKVGIHG) into the pTagRFP-N vector. In the case of GFP-53BP1, GFP is referred to as eGFP originated from eGFP-53BP1 plasmid (Addgene, 60813).

For transient transfection, the plasmids (1 µg) were nucleofected into 1 million cells with the Amaxa® Nucleofector® device (Lonza AG, AAD-1001). Nucleofector® Kit T (VCA-1002) for A549 cells and Kit V (VCA-1003) for U2OS cells were used along with program X-001 according to the product manual.

### Viability assays

We used standard live/dead dyes to assess two recognized parameters of cells viability: intracellular esterase activity (permeable Calcein AM) and plasma membrane integrity (impermeable DAPI). Leakage of expressed NLS-RFP also served as an additional confirmation of any damage to the membrane. A green-fluorescent YO-PRO-1 stain was used as an early marker of apoptosis^25,26^.

### Immunostaining of p-Trask

For standard fixation, cells were fixed for 15 minutes with 4% Formaldehyde prepared from 16% stock (Thermo Fisher Scientific, 28906) diluted 1:4 volume per volume (v/v) with 1x PBS (P5493, 10x stock) to 4% Formaldehyde, then the cells were permeabilized for 15 minutes with 1x Permeabilization Buffer (10x stock from Thermo Fisher Scientific, 1860290). For “thermofixation”, cells were quickly heated to 70 °C for 2–3 seconds using MMW radiation. The next steps were common for both types of fixation. The cells were blocked for 30 minutes with 1x Block Buffer (10x stock from Thermo Fisher Scientific, 1860291). Primary phospho-Tyr743-CDCP1 (D2G2J) rabbit antibody (Cell Signaling, 14965 S) 1:300 (v/v) diluted in 1x Block Buffer was applied to the cells and incubated for 1 hour at room temperature. Next the cells were washed 2 times with 1x Block Buffer and goat anti-rabbit IgG H&L conjugated with Alexa488 (Abcam, ab150077) diluted 1:200 (v/v) was used as the secondary antibody for 1 hour incubation together with 0.5 µg/ml DAPI at room temperature. Then the cells were washed 2 times with 1x PBS and imaged.

### Imaging

Images were acquired in brightfield, phase contrast, differential interference contrast (DIC) or epi-fluorescence modes either on a Leica DM2700 upright microscope equipped with a Leica MC120 HD color camera or on a Nikon Eclipse Ti inverted microscope equipped with a Photometrics CoolSNAP ES2 cooled CCD camera. Leica 4×/0.10 objective was used on the upright microscope and Nikon 4×/0.13, 10×/0.30, 20×/0.75 dry and 40×/1.30 oil objectives were used on the inverted microscope. The camera exposure time did not exceed 1 s for epi-fluorescence and 50 ms for all other imaging configurations. Micro-Manager or MetaMorph® software was used for image acquisition and automation. For imaging, cells were grown in Ibidi cultureware (ibidi GmbH): µ-Slide 8 Well (#1.5 polymer chambered coverslip, tissue culture treated) without (Cat.No: 80826) or with 500 µm grid (Cat.No. 80826-G500); µ-Dish 35 mm, high glass (# 1.5 H) bottom without (Cat.No: 81158) or with 500 µm grid (Cat.No: 81168). Cells were imaged live (unless specified otherwise) in Hanks balanced salt solution (H8264) supplemented with 2% fetal bovine serum (F2442). Full microscope enclosure incubator (*In Vivo* Scientific, Product No: CH.HC5.SAT) was used to control the environment. Filtersets were DAPI (Ex 340–380/em 435–485); BFP (ex 395–410/em 430–480); YO-PRO-1, Calcein, GFP, Alexa488 (ex 450–490/em 500–550) and RFP (ex 530–560/em 590–650).

### CellEraser details

The instrument is available from *In Vivo* Scientific. The vertical or Z position of the output waveguide was adjusted manually with 148–801 micrometer or in an automated mode using Z812B motorized actuator and KDC101 motor controller (all from Thorlabs). To control the distance between the output waveguide and the bottom of the cell cultureware, the waveguide was moved upwards until it touched the cultureware bottom. The touch position was detected via microscopy as image movement. Then the waveguide was withdrawn about 200 µm downwards and that distance was maintained during the calibration procedure and all subsequent experiments.

Cells exposed to elevated temperatures (50–55 °C) start to round up and lose the attachment to the substrate (similarly to cells in late stage of division). Those cells can be removed by a simple washing procedure: cell medium is removed by aspiration, Hanks balanced salt solution (H8264) is added and aspirated, and the medium is returned. Depending on the cell type, the washing step can be repeated twice to be sure that all cells are removed from the MMW treated area.

## Electronic supplementary material

Supplementary Figures and Supplementary Movie captions.

Supplementary Movie 1 : Convection induced by CellEraser: application of millimeter wave radiation to a cell suspension (RL) imaged on an upright microscope.

Supplementary Movie 2: Melting a spot in 1-HXD film using the upright microscope version of the CellEraser.

Supplementary Movie 3: Melting a spot in 1-HXD film using the inverted microscope version of the CellEraser.

Supplementary Movie 4: Automated ring melting in a 1-HXD film using the upright microscope version of the CellEraser.

Supplementary Movie 5: Automated ring melting in a 1-HXD film using the inverted microscope version of the CellEraser.

Supplementary Movie 6: “Erasing” a spot in a confluent A549 cell culture by local MMW mediated heating to 50°C for 2 seconds with washout 2 hours later.

Supplementary Movie 7: Gap closure in a confluent A549 culture after CellEraser treatment.

Supplementary Movie 8: Long-term cell fate after 50°C exposure.

## Data Availability

All data generated or analyzed during this study are either included in this published article (and its Supplementary Information files) or available from the corresponding author on reasonable request.
