# Supplementary material for: Novel millimeter-wave-based method for in situ cell isolation and other applications
Source: Sci Rep. 2018 Oct 3;8:14755. doi: 10.1038/s41598-018-32950-w (PMC6170430; doi:10.1038/s41598-018-32950-w)
Supplement: Supplementary file 1 — Supplementary Figures and Supplementary Movie captions. [file 41598_2018_32950_MOESM1_ESM.pdf]

Supplementary Information

The title of the manuscript:

Novel millimeter-wave-based method for *in situ* cell isolation and other applications

Authors:
Barney Boyce[1]
Natalia Samsonova[2*]

Affiliation:
[1] In Vivo Scientific, LLC
5 Gybe Ho Ct.
Salem, SC 26976
barney@invivoscientific.com

[2] CellEraser, LLC
15649 Century Lake Dr.
Chesterfield, MO 63017
contact@celleraser.com

[*] Corresponding author: Natalia Samsonova
Email: contact@celleraser.com

Supplementary figure legends

Supplementary Fig. 1 : Figure 1.9, page 26 from [1].

Supplementary Fig. 2 : Double spot "erasing".

Two adjacent spots in a confluent BFP-expressing A549 cell culture were sequentially heated to 50°C by local application of the MMW radiation for about 3 seconds each, demonstrating repeatability of the approach. Brightfield and epi-fluorescence images show the cell culture after the MMW treatment at indicated time points. The cells reacted to heating by rounding up and detaching from the substrate, and were removed by a media change. Coverglass bottom 35 mm dish with etched 500 µm numbered grid, 4x objective.

Supplementary Fig. 3 : Nuclear "leakage" and detachment after 50°C exposure.

U2OS cells expressing NLS-RFP were exposed to 50°C for 2-3 seconds (the entire field of view was heated by MMWs). **a**, DIC and NLS-RFP images before and 1 minute after the MMW treatment showing the exit of NLS-RFP from cell nuclei and its uniform distribution between the nucleus and the cytoplasm of every cell. **b**, DIC images of cells in A (the same field of view) at indicated time points after the MMW treatment showing the details of long-term cell reaction to 50°C as rounding and detachment from the substrate. 40x objective, the scale bar is 50 µm.

Supplementary Fig. 4 : Kinetics of GFP-tubulin nuclear entry after MMW heating of cells to 50°C.

Average fluorescence intensity of the regions of interest (bordered by red lines inside the nuclei of U2OS cells expressing GFP-tubulin) is shown as a function of time. The data were normalized at the time point before MMW application. The sharp drop in the GFP fluorescence after application of MMWs is attributed to a drop in the GFP quantum yield when the temperature rises [2]. The beginning of GFP-tubulin entry into the nucleus indicates damage in the barrier function of the nuclear envelops. Short -term (2-3 seconds) heating by MMWs results in similar kinetics of nuclear entry/exit (data not shown). 20x objective, the image width is 109 µm.

Supplementary Fig. 5 : No DNA damage after up to 60°C exposure.

A spot in a confluent culture of A549 cells expressing NLS-RFP and GFP-53BP1 was heated to 50°C (a) or 60°C (b) by local application of MMW radiation for 2-3 seconds. **a**, 50°C induced nuclear leakage to the right of the dashed line denoting the approximate border of the heated spot. In cells with NLS-RFP equilibrated between the cytoplasm and the nucleus, GFP-53BP1 remains DNA-bound as in non-treated cells. **b**, The borderline between RFP positive cells

on the left of the field of view (50°C) and RFP negative cells on the right (60°C) where the plasma membrane was compromised and RFP leaked out. GFP-53BP1 was still present in the nuclei of the leaky cells without increase in the foci number. **c**, Etoposide control showed DSBs induction seen by appearance of new GFP-53BP1 foci after application of Etoposide (three time points are shown). Coverglass bottom 35 mm dish with etched 500 µm grid, 40x objective, the scale bar is 50 µm.

Supplementary Fig. 6 : Figure 1, page 235 from [3].

Supplementary Fig. 7 : YO-PRO-1 staining after 60°C exposure.
a, An apoptotic marker YO-PRO-1 (1 µM) was added to a confluent culture of U2OS cells expressing NLS-RFP and the cells were imaged 30 minutes later. Then a spot of the culture was heated to 60°C by local application of MMW radiation for 2-3 seconds and the cells were imaged again 10 minutes later. Area where the temperature reached 60°C is shown by the dashed circles, 10x objective, the scale bar is 200 µm. **b**, Zoomed-in image of the heated spot (dashed square in panel a) overlaying NLS-RFP and YO-PRO-1 channels, 20x objective, the scale bar is 100 µm.

Supplementary movie captions

Supplementary Movie 1 : Convection induced by CellEraser: application of millimeter wave radiation to a cell suspension (RL) imaged on an upright microscope.
MMW radiation heats a narrow layer of the culture medium without heating its bulk. Convection drives the heated media upwards (perpendicular to the plane of view). The movement of the suspended cells illustrates the convectional flow of the media. 4x objective, the grid size is 500 µm.

Supplementary Movie 2 : Melting a spot in 1-HXD film using the upright microscope version of the CellEraser.
4x objective, ibidi chambered coverslip with 500 µm grid**.**

Supplementary Movie 3 : Melting a spot in 1-HXD film using the inverted microscope version of the CellEraser.
4x objective, coverglass bottom 35 mm dish. The MMW application and imaging are done sequentially.

Supplementary Movie 4 : Automated ring melting in a 1-HXD film using the upright microscope version of the CellEraser.

4x objective, coverglass bottom 35 mm dish. The internal dimensions of the waveguide are 2.540 x 1.270 mm.

Supplementary Movie 5 : Automated ring melting in a 1-HXD film using the inverted microscope version of the CellEraser.

4x objective, coverglass bottom 35 mm dish.

Supplementary Movie 6 : "Erasing" a spot in a confluent A549 cell culture by local MMW mediated heating to 50°C for 2 seconds with washout 2 hours later.

Supplementary Movie 7 : Gap closure in a confluent A549 culture after CellEraser treatment.

The initial cell-free gap (area inside the red circle) was erased by local MMW mediated heating and washout (Fig. 4 and Supplementary Movie 6). The migration into the gap was imaged over 16 hours using 10x objective, ibidi chambered coverslip with 500 µm grid.

Supplementary Movie 8 : Long-term cell fate after 50°C exposure.

The area within the red circle in a confluent culture of U2OS cells was heated to 50°C by local application of MMW radiation for 2-3 seconds. The time-lapse starts 30 minutes after heating and continues for 16 hours. Cells in the heated area are rounding and detaching from the surface. Cells surrounding the heated area are migrating inside it. Fig. 7b shows cell viability assayed at the end of the time-lapse. Ibidi chambered coverslip with 500 µm grid, the dots of the grid line were made at every 20 µm.

Supplementary Information References

1. Mehdizadeh, M. in Microwave/RF Applicators and Probes for Material Heating, Sensing, and Plasma Generation: A Design Guide. 1-33 (William Andrew Publishing, Boston; 2009).
2. Zhang, C., Liu, M.S. & Xing, X.H. Temperature influence on fluorescence intensity and enzyme activity of the fusion protein of GFP and hyperthermophilic xylanase. *Appl Microbiol Biotechnol* **84**, 511-517 (2009).
3. Henle, K.J. & Dethlefsen, L.A. Time-temperature relationships for heat-induced killing of mammalian cells. *Ann N Y Acad Sci* **335**, 234-253 (1980).

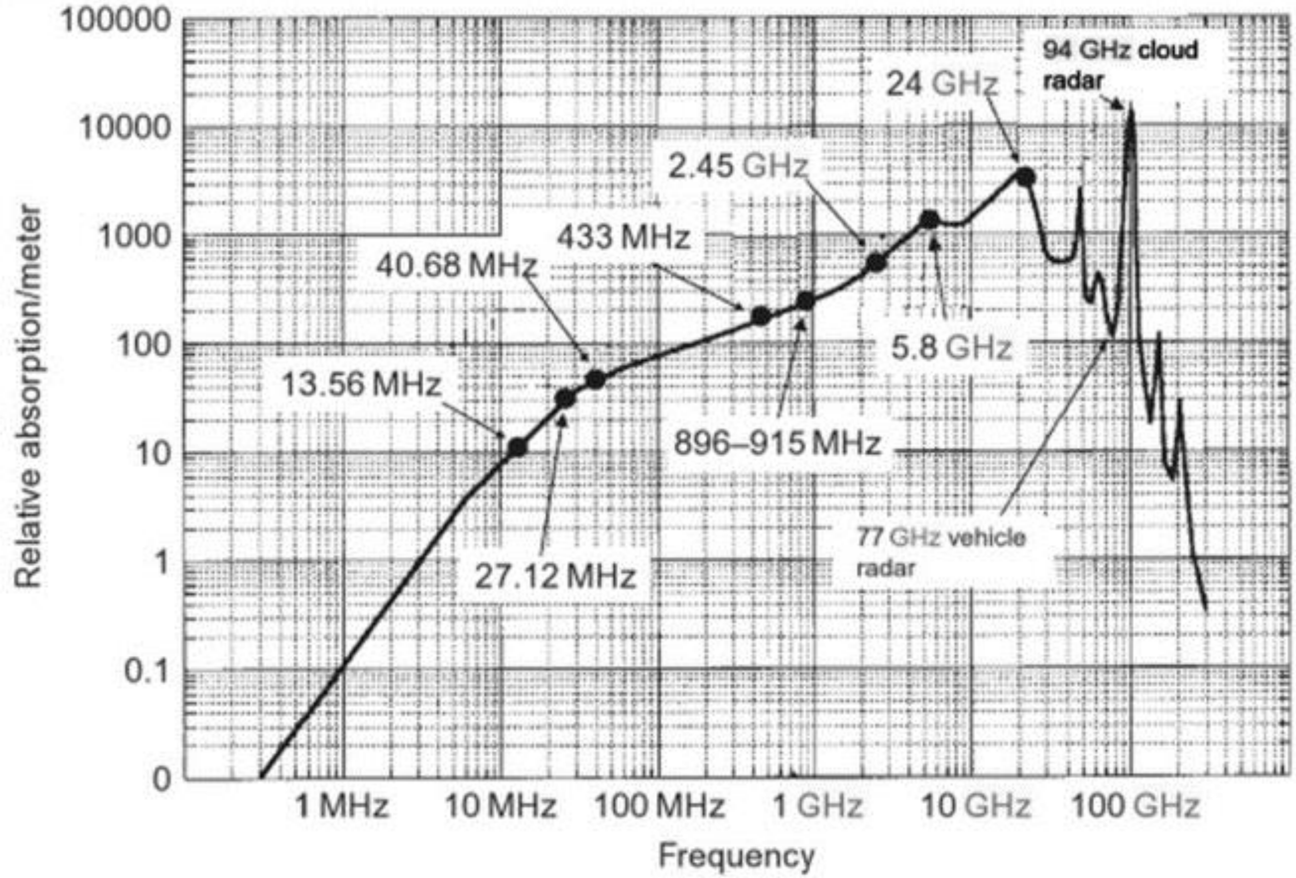

FIGURE 1.9 *Relative dissipation of electromagnetic energy by water over the frequency spectrum. There is a gentle increase in absorption with frequency. Above 20GHz there are several absorption peaks. Certain frequencies of importance in industrial, scientific, and medical applications are noted. After Jordan [40].*

Supplementary Figure 1.

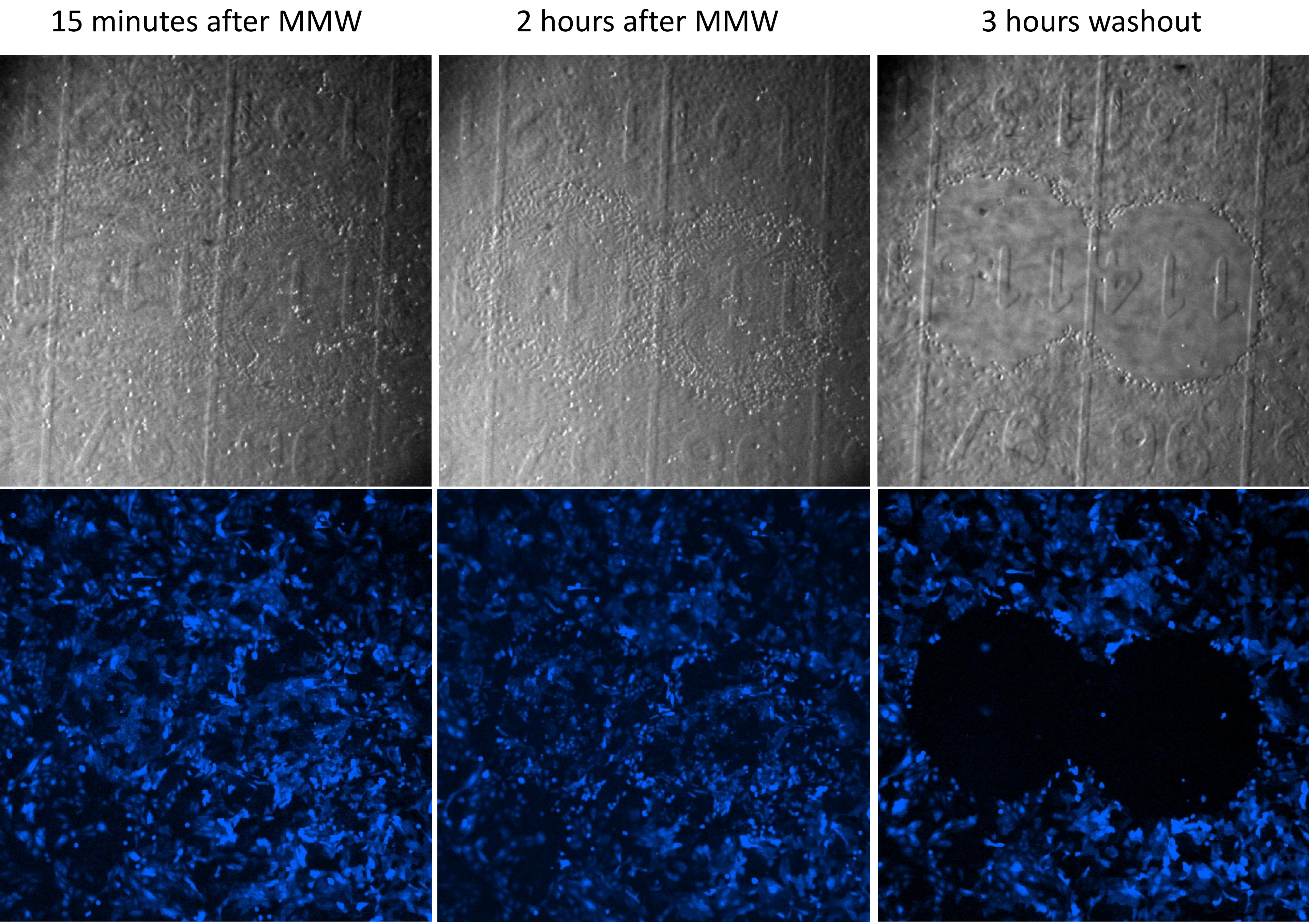

Supplementary Figure 2.

a

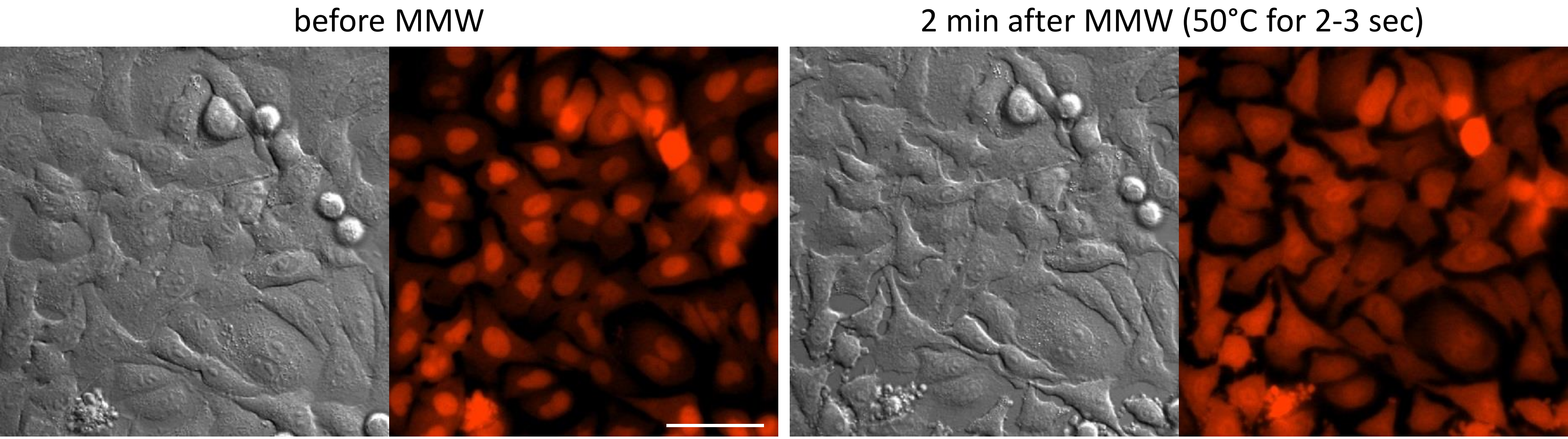

b

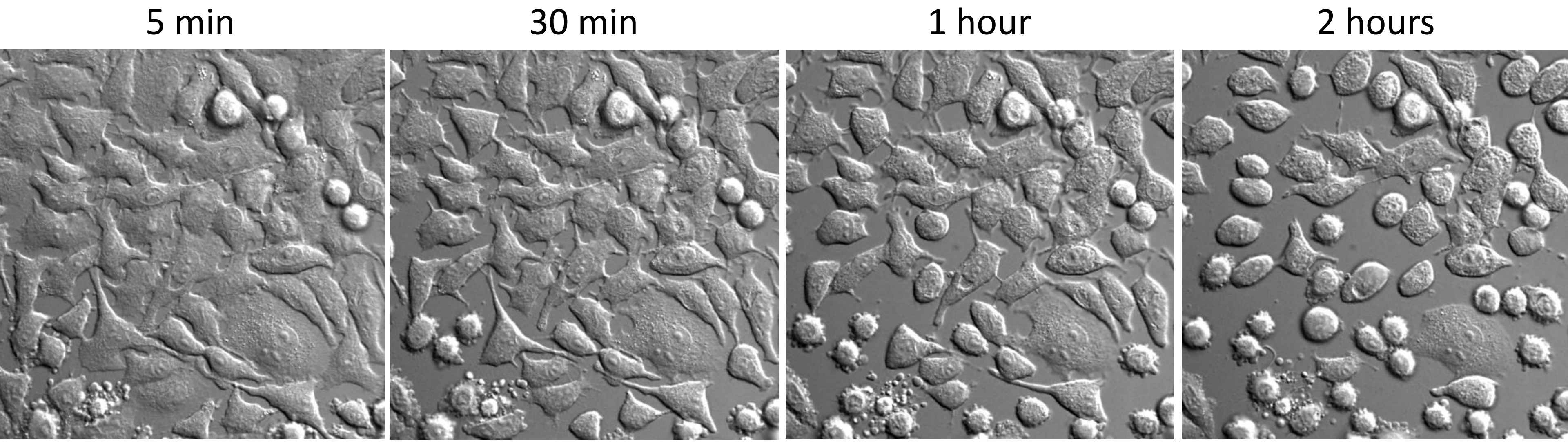

Supplementary Figure 3.

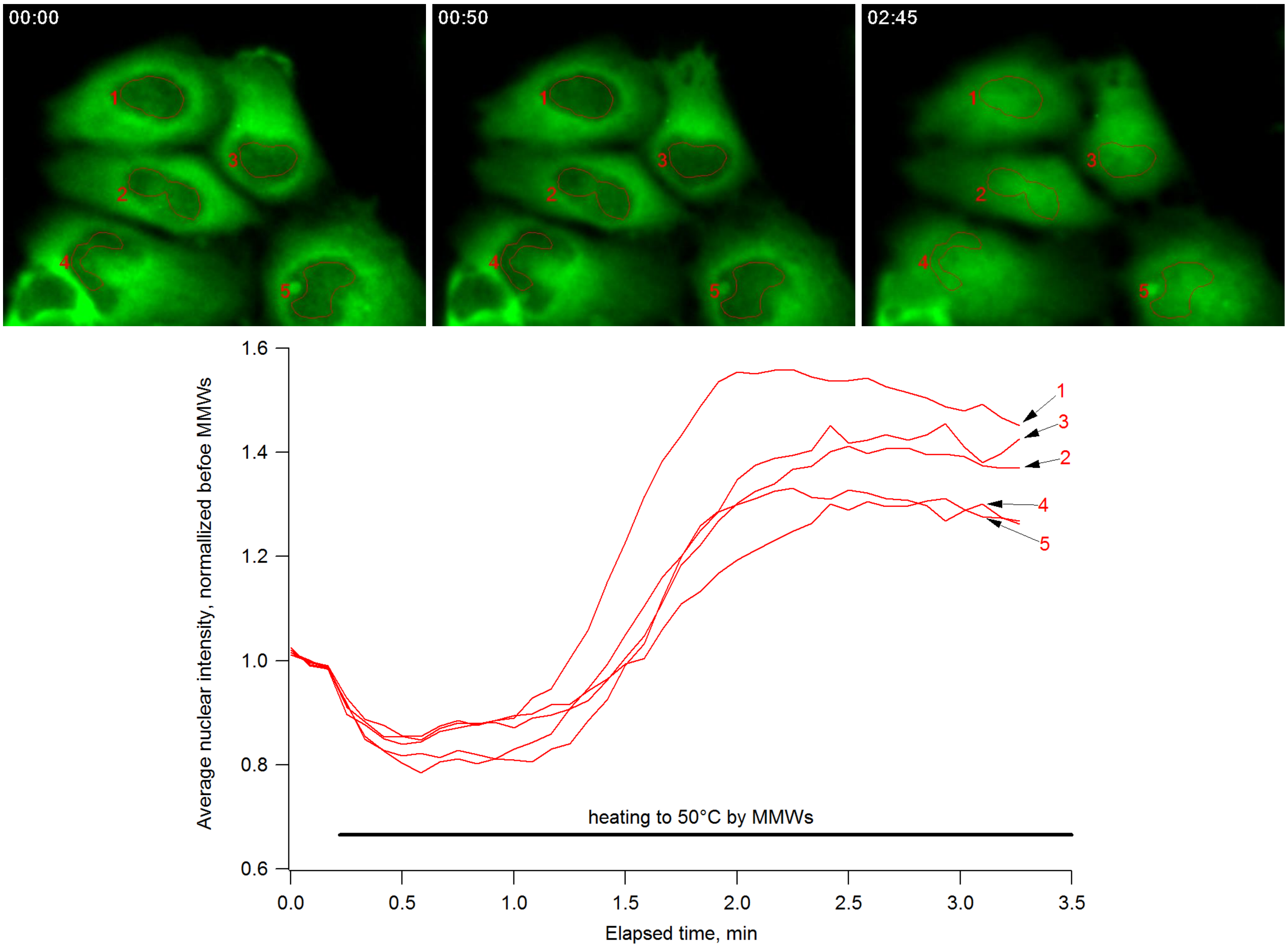
00:00
00:50
02:45
1
2
3
4
5
Average nuclear intensity, normalized befoe MMWs
Elapsed time, min
heating to 50°C by MMWs
1.6
1.4
1.2
1.0
0.8
0.6
0.0
0.5
1.0
1.5
2.0
2.5
3.0
3.5

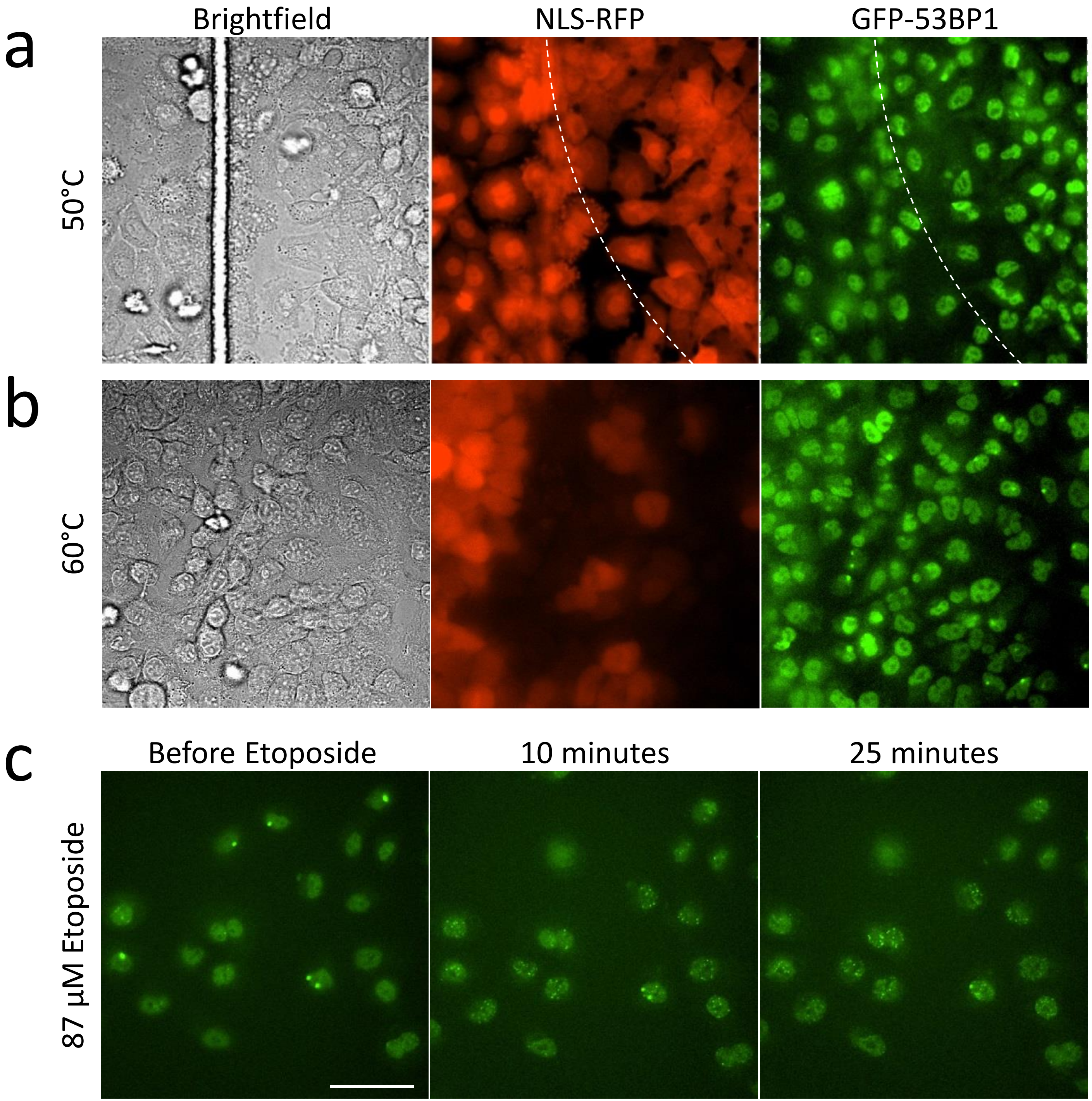

Supplementary Figure 5.

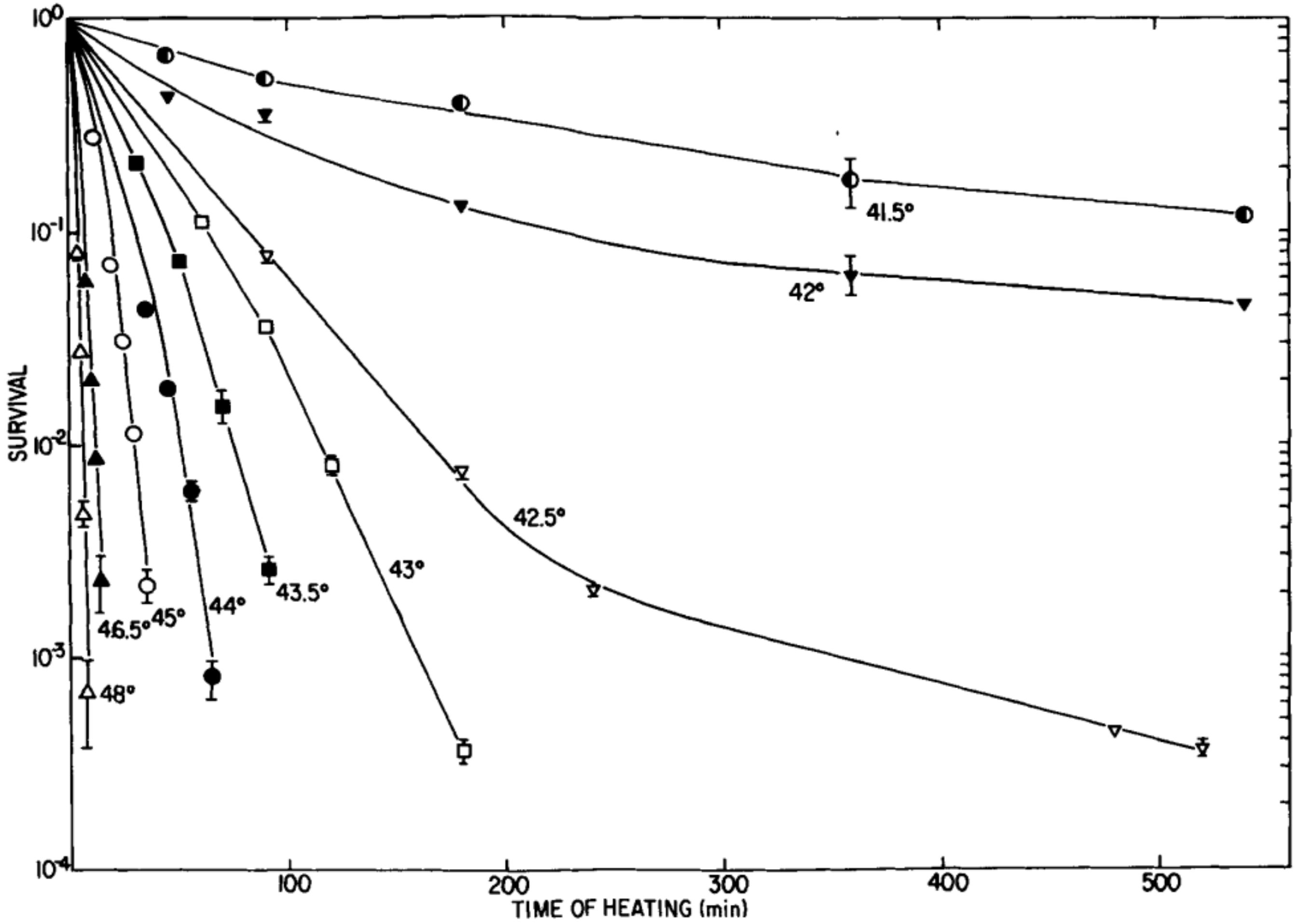

FIGURE 1. Family of survival curves for CHO cells at indicated temperatures. (From Bauer & Henle.[6] By permission of *Radiation Research*.)

Supplementary Figure 6.

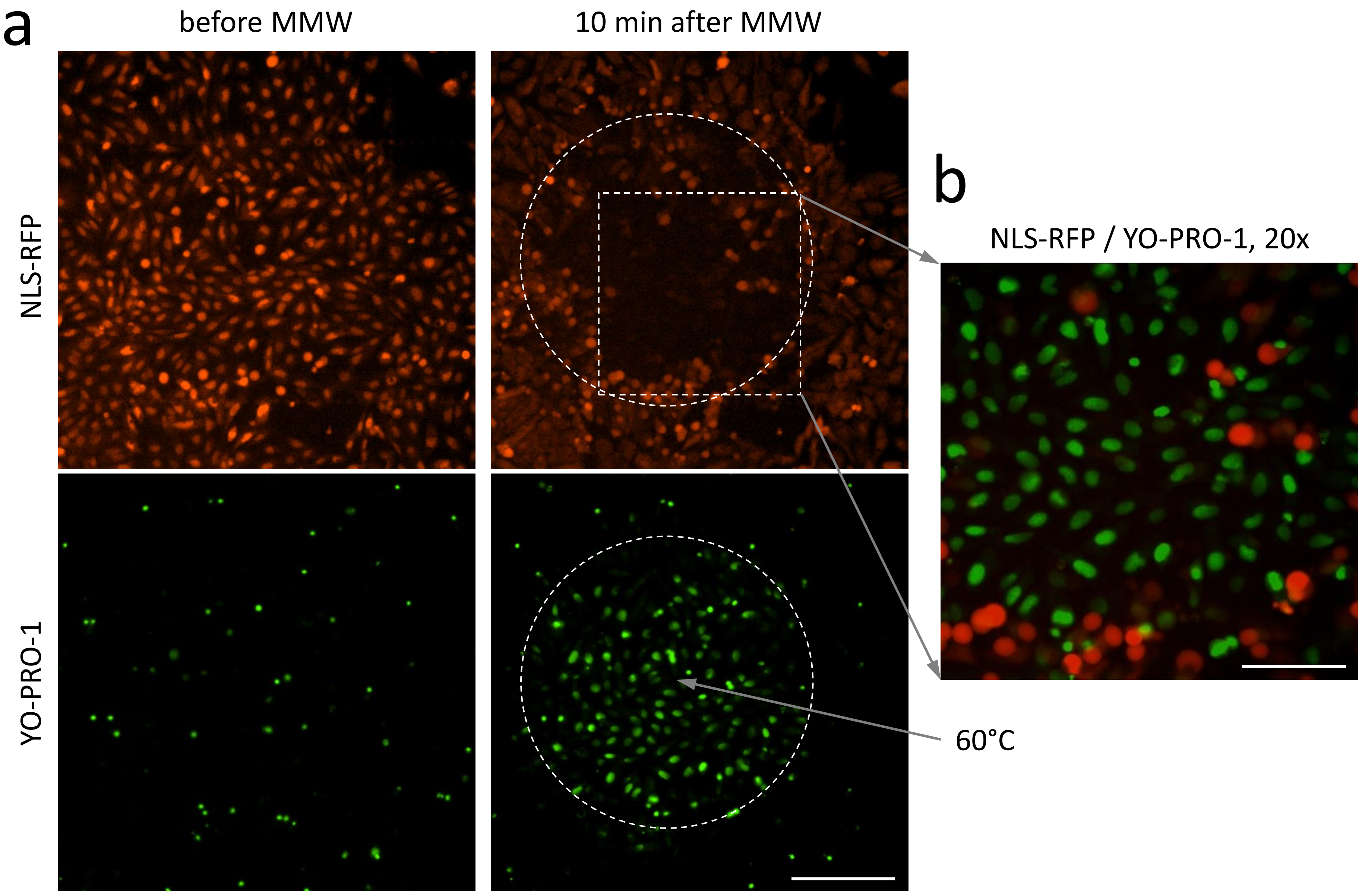

Supplementary Figure 7.
